# Virtual Genome Walking across the 32 Gb *Ambystoma mexicanum* genome; assembling gene models and intronic sequence

**DOI:** 10.1038/s41598-017-19128-6

**Published:** 2018-01-12

**Authors:** Teri Evans, Andrew D. Johnson, Matthew Loose

**Affiliations:** School of Life Sciences, University of Nottingham, Nottingham, NG7 2UH UK

## Abstract

Large repeat rich genomes present challenges for assembly using short read technologies. The 32 Gb axolotl genome is estimated to contain ~19 Gb of repetitive DNA making an assembly from short reads alone effectively impossible. Indeed, this model species has been sequenced to 20× coverage but the reads could not be conventionally assembled. Using an alternative strategy, we have assembled subsets of these reads into scaffolds describing over 19,000 gene models. We call this method Virtual Genome Walking as it locally assembles whole genome reads based on a reference transcriptome, identifying exons and iteratively extending them into surrounding genomic sequence. These assemblies are then linked and refined to generate gene models including upstream and downstream genomic, and intronic, sequence. Our assemblies are validated by comparison with previously published axolotl bacterial artificial chromosome (BAC) sequences. Our analyses of axolotl intron length, intron-exon structure, repeat content and synteny provide novel insights into the genic structure of this model species. This resource will enable new experimental approaches in axolotl, such as ChIP-Seq and CRISPR and aid in future whole genome sequencing efforts. The assembled sequences and annotations presented here are freely available for download from https://tinyurl.com/y8gydc6n. The software pipeline is available from https://github.com/LooseLab/iterassemble.

## Introduction

Salamander genomes (urodele amphibians) are amongst the largest known^1–3^. To date, no salamander genome has been fully sequenced and only limited genomic data are available^2,4,5^. Yet this group of organisms are of great interest, not just due to genome size, but also their evolutionary history, mechanisms of development and ability to regenerate^6–8^. EST sequences and transcriptomes have been generated for *Ambystoma mexicanum* (the axolotl), covering various developmental timepoints and stages of regeneration^4,9–13^. Even so, studies are limited by the lack of genome sequence, making phylogenetic comparisons difficult and many experimental approaches intractable in axolotls. For example, morpholinos, transgenesis and CRISPR-Cas9 for the study of axolotl development and regeneration have all required targeted cloning approaches and Sanger sequencing, or have focussed on transcript sequences alone^7,8,14–16^.

The emergenceof long reads, optical mapping and chromatin linkage studies can provide genomic data from more species than ever before^17^. Yet these approaches can be costly and the difficulty of sequencing and assembly scale non-linearly with genome size^18,19^. Recently, Keinath and colleagues generated 20× short read Illumina coverage of the axolotl genome^2^. Still the size of the axolotl genome, combined with the limited coverage, precluded the generation of a useful assembly.

Thus to date significant detailed genomic sequence is not available for a urodele amphibian. Limited sequencing of bacterial artificial chromosomes (BACs) have provided 24 sequences covering less than 0.01% of the axolotl genome, estimated to be 32 Gb or 10 times the size of human^5^. A key challenge for whole genome assembly is dealing with repeat sequences. Surveys of plethodontid salamanders suggest the most abundant to be members of the LTR/Gypsy retrotransposon superfamily, with repeat lengths approximately 7 kb, and so likely intractable for assembly using short read approaches^20,21^. In total, repeat sequences are estimated to make up as much as 60% of the axolotl genome^2,5^.

Given the availability of existing whole genome sequence data^2^, we reasoned that targeted local assembly around known protein-coding sequences could generate useful data characterising genic regions from the axolotl. These data could identify gene models, intron-exon structures, promoter sequences and more. These resources would be useful for those seeking to exploit common techniques to manipulate gene expression including targeted morpholinos, CRISPR-Cas9 genome editing and even ChIP-Seq.

Borrowing from genome walking as used in the laboratory^22^, we have developed a Virtual Genome Walking (VGW) pipeline using localised mapping to known transcripts to extend into surrounding genomic regions. This pipeline is exon-intron aware allowing input transcripts to be split into exons. Our methodology is similar to previous technologies designed to fill gaps within genome assemblies (such as Gapfiller and IMAGE)^23,24^, assemble flanking data (GenSeed, Tracembler)^25,26^ and assemble exons^27^. A similar protocol has been independently proposed by Aluome and colleagues, although no pipeline was made available^28^. Our approach is optimised to handle extremely large genome read sets, assemble both flanking and intronic sequence, and continue walking into unassembled genome space iteratively. We applied this method to the 20× Illumina data from Keinath *et al*., generating almost 1 Gb of assembled sequence data from the axolotl genome. We validated these assemblies against previously sequenced BACs and find our genome walked fragments closely match with 98.8% identity. We compare the resulting gene models with human finding conservation of exon/exon boundaries within coding sequences as expected.

In total we generated 19,802 gene models for the axolotl providing the largest assembled set of sequences to date. These sequences enable the inspection of more of the axolotl genome than previously possible. We note that our assembled fraction still represents only 3% of the total axolotl genome, but is a 300 fold increase in the amount of assembled genomic data available to date. The complete dataset comprising fasta scaffolds, input transcripts and annotations are available for download from figshare (https://tinyurl.com/y8gydc6n). The VGW pipeline is available to download from GitHub (https://github.com/LooseLab/iterassemble). The methods we describe are applicable to any organism with an existing transcriptome and low coverage genome reads.

## Results

### Input Transcriptome

Our previously derived transcriptome dataset^10^ was re-assembled using CLC, cd-hit and cap3 to generate 646,790 transcripts (see methods, Supplementary Fig. S1, Table 1). Using this dataset as input to our VGW pipeline would result in assembling duplicate genomic loci due to splice variants or partial transcripts. To avoid this, we identified single transcripts representing each protein-coding gene within our dataset. Transdecoder was used to detect open reading frames (ORFs) which were clustered and annotated into 23,047 protein-coding cDNAs (see methods, Table 1, Supplementary Fig. S1).

**Table 1:** Assembly metrics of the cDNA sequences.

	Number	Min Length	Max Length	Mean	N50	Total (Mbp)
Assembled cDNA	646,790	176	51,730	621	881	402
Annotated cDNA	23,047	297	51,730	2,325	4,121	53

### Genomic Read Preparation

**Figure 1:**
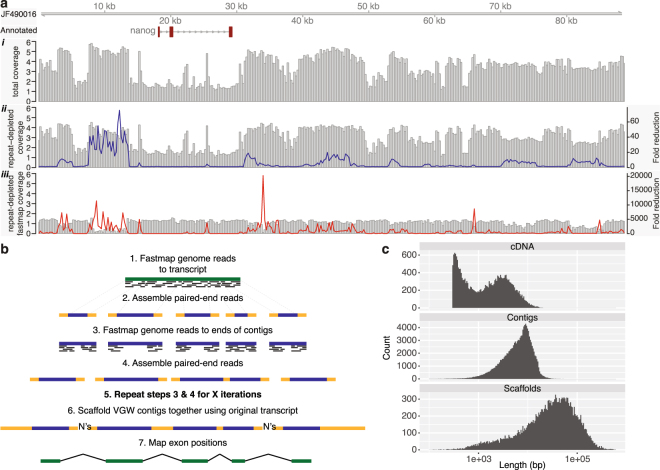
Overview of repeat depletion and VGW. (**a**) BAC JF490016 is shown with the read coverage from all reads (i), the repeat depleted reads (ii), and those that map using fastmap (iii) on a log_10_ scale. The line graphs show the fold reduction in coverage after repeat depletion (blue) and after mapping with fastmap (red). (**b**) Outline of the VGW process. (**c**) Length histogram of the input cDNAs, VGW contigs and scaffolds on a log scale.

Genomic reads for mapping to the transcriptome were publically available^2^. These data contain over 3 billion 2 × 100 base reads equivalent to 20× coverage of the axolotl genome. Keinath *et al*. were unable to assemble these data using conventional methods, but could estimate the total repeat content of the axolotl genome at between 12–20 gigabases^2^. These repeated sequences are likely to hinder both genome wide assembly, and local assemblies around transcripts, particularly when using short reads^19^. Mapping reads to a representative BAC (JF490016) revealed coverage depth exceeding 1,000,000× over some repeats (Fig. 1ai). Using Khmer^29^, we calculated median coverage of each read based on 31-mers, discarding paired reads with median coverage less than 1 or greater than 40 (47.8% of the read pairs). A further 8,957,820 (0.3%) read pairs were removed as one sequence contained an ambiguous nucleotide (‘N’). Mapping this repeat depleted read set to the same BAC demonstrates up to 70× fold reduction in coverage (Fig. 1aii). Even so, read mapping was a significant bottleneck to our pipeline (see methods). To resolve this, we indexed the reads rather than the reference and used bwa fastmap to extract super-maximal exact matches (SMEM)^30^. Mapping the repeat-depleted reads to all 24 BACs takes 5 hours with BWA MEM but only 25 minutes with BWA fastmap (30 cores, Intel(R) Xeon(R) CPU E5-2683 v4 @ 2.10 GHz). A further advantage of this approach is the increased stringency of mapping, resulting in further reductions (up to 20,000 fold) in repeat coverage with respect to the reference BACs (Fig. 1aiii).

### Virtual Genome Walking

We used these repeat depleted genome reads as input to the VGW pipeline against the unique transcript collection running 30 iterations (Fig. 1b, Supplementary Fig. S2), generating 22,794 initial genomic scaffolds. A small subset of input transcripts failed to assemble due to insufficient repeat-depleted reads mapping with a 40 base SMEM. For some no reads mapped to the transcript and we cannot exclude the possibility these are non-axolotl contaminants. The input cDNA was mapped to each genomic scaffold using GMAP^31^ and any unmapped contigs removed. Using the new intron sequences we identified additional redundant transcripts and merged the resulting VGW scaffolds (see methods). After processing, our final VGW dataset comprised 19,802 scaffolds in 128,833 contigs (Table 2, Fig. 1c). The longest scaffold, at 585,289 bp, is derived from a 13,848 bp transcript and consists of 79 exons mapped over 63 contigs and is likely orthologous to the human VPS13D gene. Our contig N50 at 9,306 bp compares favourably with Illumina only whole genome assemblies from other large genomes^32^, although our contigs only include genic regions. The scaffold N50, at 82,884 bp, is effectively equivalent to a partially sequenced BAC library.

**Table 2:** The VGW assembly metrics.

	Number	Min Length	Max Length	Mean	N50	Total (Mbp)
Contigs	128,833	100	52,850	7,313	9,306	942
Scaffolds	19,802	119	585,289	47,578	82,884	942

Scaffold metrics exclude the artificially inserted 500 ‘N’ gaps between contigs.

**Figure 2:**
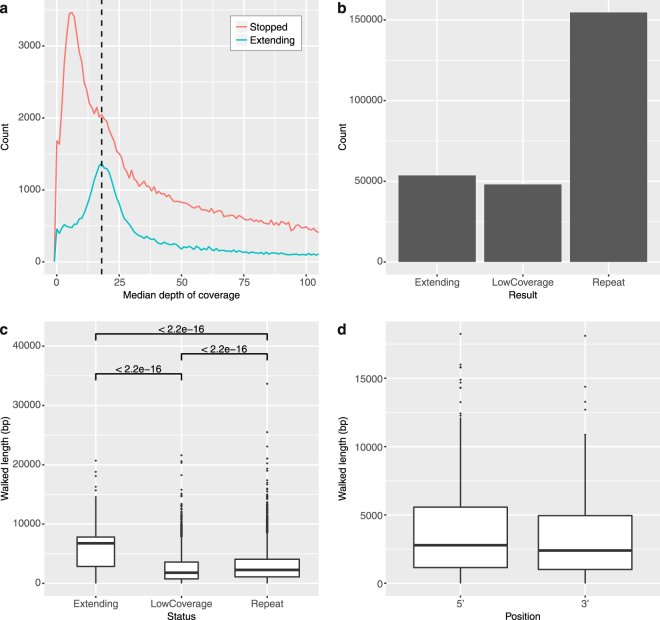
Most contigs stopped extending due to repeats. (**a**) Shows the median depth of coverage over the last 60 bp of each contig, the results are separated based on whether the end of that contig continues to extend in iteration 31. The mode depth for extending contigs (dashed line) is used to divide the others into low coverage and repeats. (**b**) Most contigs stopped extending because of repeats, defined by a high median coverage. (**c**) The length walked from the nearest mapped exon is shown according to the contig status. (**d**) The length walked from the outermost exons is shown, irrespective of whether the contig had stopped extending.

We ran VGW for 30 iterations to investigate algorithm performance. Given the prevalence of high copy long repeats in the axolotl genome, we hypothesised repeats would be the most common cause of VGW failing to extend a contig. Alternatively, given only 20× coverage, insufficient coverage of specific regions might prevent a contig from extending. To investigate this we ran an additional iteration identifying transcripts still extending (see methods). 14,179/22,794 (62.2%) transcripts that began extending were still being processed by iteration 30. However, the majority of contigs within each extending transcript (98,413, 76.4%) had stopped extending by iteration 30. To distinguish contigs stopped by low coverage compared with repeats, we calculated the coverage depth (mode) at the ends of extending contigs compared with stopped contigs (Fig. 2a - see methods). As anticipated, in the majority of cases VGW stopped extending due to repeat sequences (76.3%, Fig. 2b).

We mapped transcripts back to the genome fragments to determine the length walked from the closest exon (Fig. 2c). Contigs still extending had walked significantly further from the closest exon than those stopped due to repeats or low coverage. However, a subset of contigs marked as extending are shorter than expected. Two observations may explain this: some contigs fluctuate in length from iteration to iteration whilst others only extend by a small number of bases. The median length walked from the nearest exon for all contigs still extending is 6,717 bp, thus VGW is extending the end of each contig by approximately 220 bp each iteration. Examining the distances walked from the outermost exons of each gene model demonstrates we assembled 2,607 bp on average (median) of flanking DNA either side of each gene (Fig. 2d). We thus assume the majority of promoter sequences will be included within the VGW scaffolds as human promoter binding sites are typically within 30–300 bp of the transcription start site^33,34^.

### Classifying Repeats

**Figure 3:**
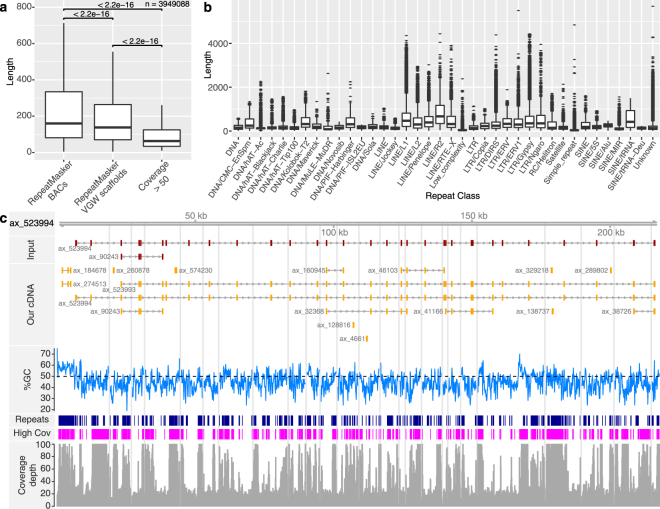
Axolotl Repeats. (**a**) Boxplot showing the difference in repeat lengths between those identified by RepeatMasker on either the BACs or VGW scaffolds, and those with coverage greater than 50×. (**b**) The length of repeats identified within the VGW scaffolds, separated by repeat class. Only repeat classes that were identified more than 500 times are shown. (**c**) The VGW output for ax_523994, this gene has the longest repeat identified of 5,695 bp. The repeats masked are shown in dark blue, the regions with coverage greater than 50 are shown in magenta. The coverage depth, up to a maximum of 100, is shown at the bottom. Contig breaks are shown by the grey bars.

Given the prevalence of repeat sequences, we generated a repeat library from all available axolotl genomic data, including the available BAC sequences (see methods). Using this library of 2,103 classified repeats, RepeatMasker masked 34.90% of the VGW scaffolds. The majority of masked repeats were classified as unknown (19.96%), while 7.98% were identified as LINE and 5.27% as LTR elements. The mean length of masked repeat sequence was 225 bases, shorter than the mean length of masked repeats on the BACs (283 bases), but longer than the peaks of high coverage (Fig. 3a). The longest repeat masked in the VGW scaffolds is 5,695 bp (unknown class), suggesting that in some cases we have walked further across, presumably diverged, repeats than anticipated (Fig. 3b). Only 12.4% of masked repeats were located within 60 bp of the end of a contig, suggesting that the majority of repeats masked were walked through to completion. This may be due to the high stringency of BWA fastmap negating problems of high coverage in divergent repeats, as suggested in Fig. 1a.

The most common repeat identified within the VGW scaffolds was unknown and named ‘rnd-1_family-16’. This was masked 15,251 times with a consensus sequence of only 155 bases. We believe this repeat is likely derived from a small non-coding RNA or retroposon similar to other examples reported in salamanders^35^. In agreement with the observations of Keinath *et al*.^2^ the next most common repeat outside the unknown class was a LINE/L2 element (1,488 bp consensus), not an LTR/Gypsy repeat as suggested by Sun *et al*.^20^. Figure 3c shows ax_523994, which contains the longest masked repeat; regions with coverage greater than 50 are also shown. Peaks of coverage and masked repeats often appear at the ends of contigs suggesting these repeats caused the VGW to stop extending.

### Assessing scaffold quality

**Figure 4:**
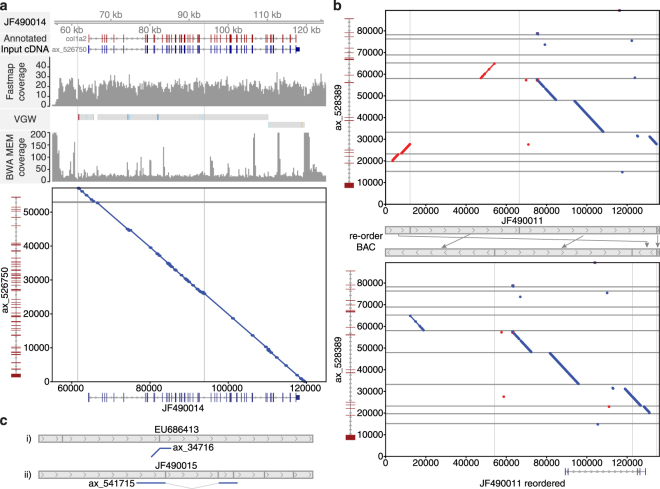
Comparing the VGW scaffolds and BAC sequences. (**a**) A high quality, long VGW scaffold maps to BAC JF490014. The intron exon structure mapping to this BAC is shown along with read depth coverage plots. The VGW row marks in grey 100% identity compared with the BAC through both exon and intronic sequence. Specific mismatches are highlighted in colour. Horizontal and vertical grey bars represent gaps within the VGW and BAC assembly respectively. Exact matches of 20 bp or more are shown in red and blue in the dot-plot, representing the forward and reverse direction respectively. (**b**) The dot-plot shows that some of the JF490011 BAC fragments are in a different orientation to the VGW scaffold. After re-orientating the BAC fragments according to our assembled scaffold, we are not only able to map the transcript but also show how we walked across one of the BAC gaps. (**c**) Shows cartoon examples of two scaffolds that are contiguous with the BAC, yet the BACs are most likely mis-assembled. Dot-plots for these two comparisons are shown in Figure S3.

Before analysing the VGW scaffolds in detail, we sought to measure the quality of our assembly. We compared the VGW scaffolds against the 24 publicly available BAC sequences^5^ and were able to identify 16 BACs with corresponding VGW scaffolds (see methods, Table S1). The VGW scaffolds mapped with a mean identity of 98.8% across their entire lengths, the same mean identity as the cDNA sequences map to the BACs. This indicates that the genome walked introns and surrounding sequence are similar quality to transcripts assembled using conventional methods. Fourteen VGW scaffolds mapped contiguously to the BACs. The longest VGW scaffold (illustrated in Fig. 4a) is comprised of two contigs and contains a complete gene model annotated as collagen type 1 alpha 2 chain (COL1A2). There are 52 mapped exons in this gene; the longest intron is 5,064 bp and has been completely bridged by VGW. In addition we close a previously un-assembled gap in the reference BAC. This example also illustrates VGW contigs stopped extending at both regions of low coverage and high-copy repeats. Interestingly, the 5′ contig could not be extended beyond the same point as a gap in the BAC, suggesting that this region of the genome presents a significant challenge to assembly irrespective of method.

Two non-contiguous scaffolds mapped to fragmented BACs (Fig. 4b). Here the VGW scaffold maps contiguously to each fragment but the fragments are incorrectly ordered and orientated with respect to the VGW scaffold. One contig maps to two non-contiguous BAC fragments. Although the transcript aligned to the BAC with 99.4% identity, GMAP failed to map the gene model with correct intron-exon boundaries. After re-orienting and ordering the BAC fragments based on the VGW scaffold, GMAP was able to exons. Originally this BAC was unannotated presumably due to the mis-assembly. The second VGW scaffold that appeared non-contiguous also suggested an incorrectly assembled BAC (Table S1). Furthermore, two other VGW scaffolds, although contiguous, indicated the respective BACs were incorrectly assembled (Fig. 4c, Supplementary Fig. S3). One shows the VGW contig extending beyond the BAC fragment, yet that fragment is positioned within the middle of the BAC. In the other case, a single VGW contig skips a central fragment again suggesting incorrect BAC ordering.

It should be noted that not all contigs within these scaffolds are able to map to the BACs. These additional contigs are derived from exons that are beyond the BAC sequence. In ax_528389 (Fig. 4b) 7 additional exons were identified upstream of the reordered BAC, and 2 exons downstream, all of which have generated genomic contigs. Across all 16 VGW scaffolds being compared to the BACs, only 7 contain the same number of exons as on the BAC (Table S1). This highlights how VGW is able to assemble and scaffold genomic fragments containing a single gene, irrespective of the distance between exons.

### Examining Introns

**Figure 5:**
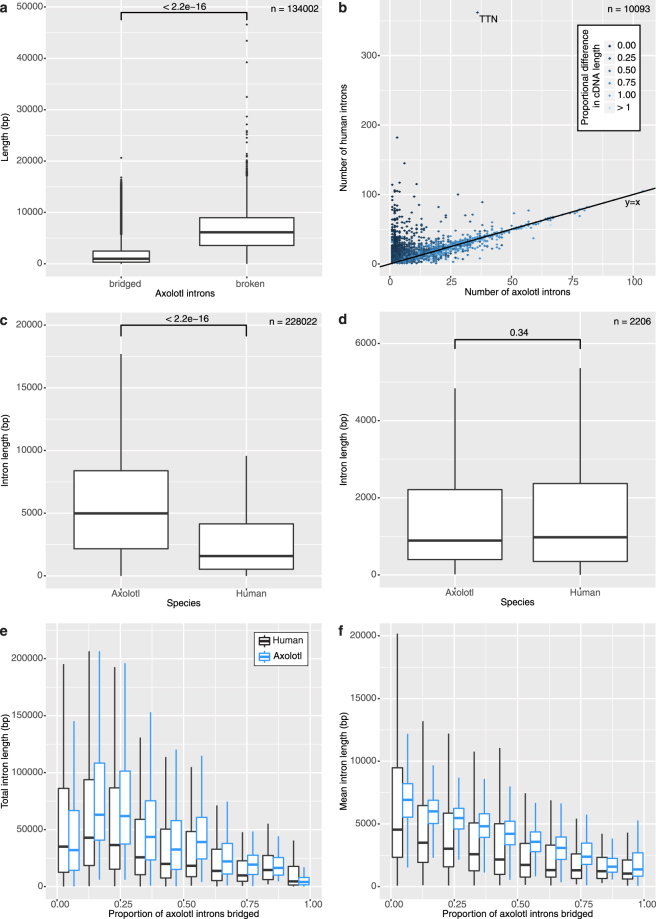
The Axolotl introns. (**a**) Boxplot of axolotl intron lengths comparing those we can bridge, and those we cannot. (**b**) Number of introns in axolotl and human, plot is coloured by the ratio of axolotl to human CDS lengths. (**c**) Intron length distributions in axolotl and human. (**d**) Intron length distribution in axolotl and human for completely bridged genes. (**e**) Total intron length per proportion bridged in axolotl (blue) and human (black). (**f**) Mean intron length per proportion bridged.

To analyse intron lengths within our gene models, we identified transcripts mapping in a single path to their corresponding VGW scaffold. 15,364 of 18,448 paths had more than one exon mapped to the genome. We removed 184 gene models from our analysis as an intron contained multiple contigs, and so the length was unreliable. The 15,180 gene models remaining contained 134,002 introns, of which 35,300 (26.34%) were completely assembled by VGW. 689 gene models (4.54%) were mapped to a single contig, with 378 of these containing only a single intron. As expected, completely assembled introns are significantly shorter than those that remain unassembled (Fig. 5a, Supplementary Fig. S4). This suggests high-copy conserved repeats are present within long intron sequences in axolotl which cannot be walked across. Supplementary Figure S5 shows two examples of VGW fragments, the first shows one of the longest introns we are able to assemble (16,818 bp, ax_530571 orthologous to human LRIG3). The second shows the longest completely assembled gene model that maps to a single contig of 43,023 bp (ax_572447, orthologous to human MYT1).

Based on analysis of relatively few BAC sequences, Smith *et al*. proposed that axolotl introns are approximately 10 times larger than in other vertebrates, consistent with the overall genome size expansion^5^. We therefore aligned the 15,180 transcripts described previously against human protein-coding genes using BLASTn. 12,604 genes matched with an e-value of less than 1e-10, of which 10,093 were unique ensembl IDs (see methods). This identified 3,857 (38.21%) axolotl transcripts that have the same number of introns as in human, with 8,613 (85.34%) showing a small difference of +/− 5 introns (Fig. 5b). As expected from a potentially incomplete transcriptome assembly, many genes have more introns in human than axolotl. Exon boundaries appear conserved with human, with over 65% of the axolotl boundaries present within 10 bp of the human exon-intron boundary (see methods, Supplementary Fig. S6). Although less conserved than *Xenopus*, we believe the difference is due to small inaccuracies in GMAP defining the exon positions.

Although many axolotl introns are incomplete, they are still larger (median 3×) than the equivalent human introns on average (Fig. 5c). However, fully bridged genes show no significant difference in intron length (Fig. 5d). Shorter genes that we are able to bridge in VGW also tend to be shorter in the human genome (Fig. 5e,f). There are some cases where gene models are significantly expanded with respect to the human genome. For example, ax_517324 has a scaffold length in excess of 450 kb even though almost none of the introns are bridged (Supplementary Fig. S7). Yet its ortholog in human (ENSG00000123384, LRP1) only covers 100 kb. However, ax_576019, for which every intron is completely bridged, has a total intron size of only 3 kb. The ortholog in humans (ENSG00000143333, RGS16) differs in total intron size by only 49 bp, although the individual introns do vary. Both of these example genes contain the same number of exons in Axolotl as in Human. Overall, our results suggest that intron size is gene dependent and that the genome size expansion is not uniform across the genome.

### Transcript mappings

By mapping additional RNA-seq datasets to each VGW scaffold, we can distinguish transcript variants from gene copies (Supplementary Fig. S8)^12,13^. Similarly, genes thought to have multiple copies in axolotl can now be confirmed using genomic sequence, such as Nodal and Brachyury (Supplementary Fig. S9 and Fig. S10)^7^. As only one copy of each gene is present in humans, both axolotl Nodal genes are annotated as the same gene in the Jiang dataset, as are both copies of Brachyury. This demonstrates the increase in resolution obtained by mapping against VGW scaffolds.

A subset of transcripts map to more than one VGW scaffold. In most cases, mappings were low identity and likely derived from simple repeats or regions of low complexity. Occasionally one or more transcripts mapped with high identity outside of the known exon positions. Furthermore, in 248 instances the two VGW scaffolds shared identity across this region, suggesting these genomic fragments might be linked. The most likely cause is fragmented transcripts, as low level expressed transcripts may not have been completely assembled in the transcriptome. To assess this, we analysed the Jiang and Bryant datasets^12,13^, looking for transcripts which overlapped exons on both VGW scaffolds. This identified 210/248 were likely derived from the same gene. In one case, a single transcript from the Jiang dataset mapped to exons on three overlapping VGW scaffolds. We were able to assemble these into a single scaffold using CAP3 (Supplementary Fig. S11) demonstrating how VGW has walked between these fragmented transcripts.

To further analyse the remaining 38 pairs of scaffolds, we identified their most likely human ortholog using BLASTn. Only 20 pairs found a human ortholog for both axolotl transcripts with an e-value less than 1e-10. Exactly half of these were also derived from fragmented transcripts from a single gene, identified either through non-overlapping BLASTn HSPs to the same human gene, or manual inspection. Of the remaining 10 pairs of linked VGW scaffolds, one appears to be a repeat as both transcripts map within repeat masked sequence. Two of the pairs may be mis-assemblies, as the linked contigs contain only a single exon that is not represented in either the Bryant or Jiang transcripts. Furthermore both of these axolotl genes contain an additional exon compared to their human ortholog. This could be a case of transcript mis-assembly leading to an incorrect VGW scaffold that appears to contain two genes. This leaves 7 pairs of axolotl scaffolds that appear to be syntenic, as they contain two distinct genes. This is corroborated by the human orthologs, 5 of which share synteny, indeed they are the closest genes to one another in the human genome (Table 3). The mean inter-gene distance between these human genes is 1,130 bp, although 3 genes overlap and therefore have a distance of 0. The mean inter-gene distance in axolotl is 1,722 bp, and is not significantly different to the human distance (p = 0.0625; Wilcoxon signed-rank test). The remaining two genes are not syntenic in Human, Chicken or *Xenopus*; without further validation of these VGW scaffolds it is not possible to know if these are genuinely syntenic in axolotl.

**Table 3:** Syntenic genes that VGW was able to assemble.

Gene 1	Human 1	Gene 2	Human 2	Human distance	Axolotl distance
ax_534869	XRN2	ax_58426	NKX2-4	5540	6391
ax_536903	ANAPC2	ax_10551	SSNA1	110	536
ax_548767	GALNS	ax_582117	TRAPPC2L	0	794
ax_522741	RETREG3	ax_550041	TUBG1	0	496
ax_538502	BCS1L	ax_520559	ZNF142	0	393

Distances of 0 are shown for genes with overlapping exons.

One example of a syntenic gene is shown in Supplementary Fig. S12, the VGW scaffolds of ax_538502 and ax_520559 are assembled together using CAP3. Two distinct genes map to this single scaffold, orthologous to BCS1L and ZNF142 respectively. Both axolotl genes share the same number of exons as in human, and are only separated by 393 bp. This further suggests that the axolotl genome size increase is not uniform.

### Chromosomes 13 and 14

Finally, we looked at whether the chromosome capture sequencing of chromosomes 13 and 14 could be used to distinguish which gene models are situated on these chromosomes^2^. To do this we associated 615 VGW fragments with the known linkage groups (LG) based on at least two primers aligning to our transcripts^3^. We then compared the coverage depth across exons of the 40 linked genes on AM13 (LG15 and LG17) and the 19 AM14 (LG14) genes with those on the other linkage groups. Unfortunately, the appropriate SRA files were not annotated with chromosome of origin. Therefore to distinguish from which chromosome the reads were derived, we first compared each uploaded SRA file and grouped them accordingly (Supplementary Fig. S13). We then re-mapped the data using these grouped files to improve coverage. Although the chromosome capture sequencing was at low coverage, we were able to enrich for genes present in the chromosome 13 and 14 linkage groups (Supplementary Fig. S13).

We then mapped these reads to all VGW scaffolds, and calculated the coverage depth across the exon positions. By using parameters identified for the linkage group genes (Supplementary Fig. S13), we were able to isolate 1,708 axolotl VGW scaffolds on AM13, of which 596 transcripts had a blast result in human (e-value < 1e-10, 546 unique Ensembl IDs). We were able to isolate 1,397 VGW scaffolds on AM14, corresponding to 399 human genes, of which 368 are unique. We note that the original number of axolotl genes isolated is higher than expected for the two smallest chromosomes as if all 14 chromosomes had an equal number of genes we would only see 1,414/19,802 genes per chromosome. Furthermore, there are a large number of genes with no BLASTn alignment to human protein-coding genes, which suggests the additional axolotl transcripts may be transposases present on more than one chromosome. Indeed, 656 of the isolated axolotl transcripts are shared between AM13 and AM14, only 21 of which have a human BLASTn match.

**Figure 6:**
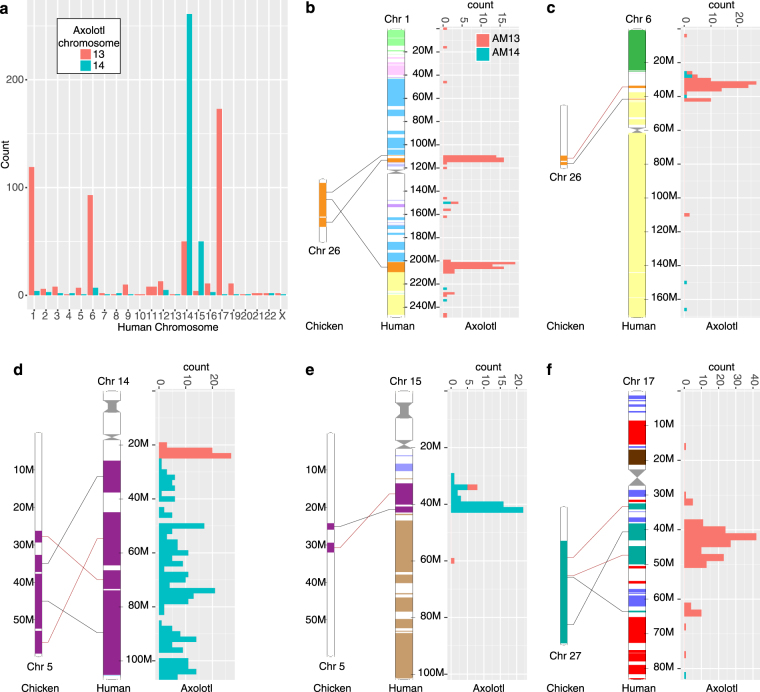
Synteny between axolotl, human and chicken. (**a**) The number of axolotl genes on AM13 and AM14 orthologous to genes on human chromosomes. The location of the orthologous genes are shown, along with synteny between that region in human and chicken (Ensembl) for human chr1 (**b**), chr6 (**c**), chr14 (**d**), chr15 (**e**) and chr17 (**f**).

By analysing the location of the human orthologs to the unique AM13 and AM14 axolotl genes we are able to compare synteny between axolotl and human. The AM13 genes were associated with human chromosomes 17, 1 and 6 while the axolotl AM14 genes were associated with chromosomes 14 and 15 (Fig. 6). The regions on these human chromosomes are highly syntenic with chicken chromosomes 26, 5 and 27; all of which were previously highlighted as being syntenic with axolotl chromosomes 13 and 14^2^. Therefore, the chromosome capture sequencing reads can be used to classify axolotl VGW scaffolds consistent with direct assembly methods.

### Comparison to whole genome assembly

While writing up this work, a whole genome assembly utilising Keinath *et al*.’s previous paired-end reads and a new mate-pair library was released as an unpublished queryable BLAST database (www.ambystoma.org). The assembly consists of 21 Gb in 21 million scaffolds with an N50 of 27,236 bp. Although a complete comparison is not possible due to limited access, we used BLAST to investigate 200 randomly chosen VGW scaffolds and extracted all sequences with an e-value of 0 and bitscore greater than 1000. We specified this level of stringency to limit spurious matches caused by repeats. Nevertheless, 185 of our VGW scaffolds extracted 3,531 whole genome scaffolds. We identified and removed 2,040 of these potential BLAST matches as mapping to repeats because they either mapped to multiple VGW scaffolds or contained overlapping HSPs to the same VGW scaffold. To better understand the remaining 1,491 whole genome scaffolds (Table 4) we split the sequences on gaps of at least 100 ‘N’s and identified these as contigs. Considered either as scaffolds or contigs, the mean and N50 values are shorter than our VGW sequences. Over these genic regions the transcriptome provides more information than the mate-pair library alone. 24% of our VGW contigs are represented by more than one whole genome scaffold, as is apparent from the large number retrieved by BLAST.

**Table 4:** Assembly metrics for 200 random VGW scaffolds and their corresponding whole genome (WG) scaffolds.

	Number	Min	Max	Mean	N50
VGW - scaffolds	200	638	320,175	50,759	86,249
VGW - contigs	1395	144	28,192	7,277	9,146
WG - scaffolds	1,491	565	370,994	25,104	39,788
WG - contigs	13,671	14	83,208	2,738	4,723

Only 119 whole genome scaffolds mapped to more than one of our VGW contigs. As an example we show a VGW scaffold of 117 kb aligned against a whole genome scaffold of 74 kb (Supplementary Fig. S14). The putative exon positions were identified by mapping the VGW transcript against both scaffolds. The whole genome scaffold is contiguous with our assembly. In total, 111 (93.3%) whole genome scaffolds are contiguous with our VGW scaffolds. These whole genome scaffolds bridged 251 contig breaks, however, only 19 (7.6%) were completely assembled without gaps of more than 100 ‘N’s. Supplementary Figure S14 also shows that most of the gaps in the VGW scaffold are also represented with ‘N’s in the whole genome scaffold. This suggests that the axolotl repeats that stop VGW cannot be resolved using mate-pair assembly alone.

## Discussion

Here we demonstrate how low coverage Illumina data can be used to generate gene models even when the raw reads themselves cannot be assembled by conventional methods. Using the 20× Illumina reads generated by Keinath *et al*. we have assembled almost 1 Gb of the axolotl genome^2^. These sequences are not random samples of the genome, rather they represent gene models for over 19,000 genes. This is a unique and valuable resource for the field that will permit studies on evolutionary genomics and the use of genomic molecular biology methods on a salamander model species. As well as building gene models and assembling intronic sequence, we have been able to walk on average 2 kb upstream and downstream of each transcript. In principle this data set should include the majority of promoter sequences for each transcript and could enable the use of ChIP-seq methods against promoters in the future.

Using the dataset we have generated a new axolotl repeat library containing 2,103 consensus sequences. Our VGW algorithm allowed us to walk through an unexpectedly high number of repeats. Although the majority of these were short, we were able to assemble repeats >1 kb. In total, over 30% of our VGW scaffolds were identified as being repeat sequence; we anticipate a higher proportion of repeats outside of the genic regions as suggested in Fig. 1a.

We show that the genome gigantism observed in salamanders does not appear to be uniform across genic regions in the axolotl genome. Indeed, gene model lengths in axolotl correlate with their orthologous lengths in human. Genes that we have completely assembled show no significant increase in overall intron size compared to their human orthologs. Where introns cannot be bridged, the minimal expansion is 3× the equivalent distance in the human genome. The upper estimate on expansion is at least 10× based on the observations of Voss *et al*.^3^. Furthermore, in a handful of cases we can walk between axolotl genes, suggesting that intergenic regions are not uniformly expanded either.

We have utilised additional resources built for the axolotl community to map some of our assembled scaffolds to chromosomes 13 and 14 showing that even low level coverage of individual chromosomes is sufficient for these classifications. Therefore, continued short-read sequencing of chromosome capture, combined with the known linkage map, could allow further classification of both our VGW scaffolds and whole genome assembly scaffolds in the future.

Whilst writing up this work, a new Illumina based assembly employing mate-pair libraries was released. This data set, representing almost 21 Gb of the axolotl genome is currently only accessible by blast searching and has a scaffold N50 of 27,236 bases (see www.ambystoma.org). We have tested a subset of these with super contigs released by Voss and colleagues and show that our contiguity agrees well with whole genome assemblies. Interestingly, we find that some regions cannot be assembled through in either data set, confirming that mate-pair short reads alone will be insufficient to dramatically improve the assembly contiguity. Rather a long read strategy is likely required incorporating either Pacific BioSciences and additional technologies^17^ or ‘ultra-long’ reads from the Oxford Nanopore platform^36^. It will be interesting to determine if VGW using more comprehensive read sets including mate-pairs will enable higher quality gene models than simple whole genome assembly.

The VGW method itself has general utility for analysing genomes with low coverage read sets available. Whilst cost is not typically a limiting factor for traditional model organisms and human genomes, it is often a concern to those working on less well studied models. Such models are often found to have larger genomes, which substantially increases the cost of sequencing. Such genomes also tend to be repeat rich, requiring elaborate library strategies to resolve on conventional short read platforms. We have shown here that useful genomic data can be recovered in such circumstances from limited coverage of a reference genome. Indeed, for many common genomic approaches a whole genome is not absolutely necessary, rather a comprehensive targeted approach can provide as much benefit as the entire genome.

## Methods

### Transcriptome sequencing and Assembly

The axolotl RNA was isolated from oocytes and a range of developmental stages^10^. Each library was initially assembled using CLC (QIAGEN Bioinformatics) with the default parameters, we then merged each library by clustering with cd-hit and assembling each cluster using CAP3^37,38^. To identify protein coding regions, Transdecoder was first run to identify all long putative open reading frames, these were then blasted against a vertebrate specific protein nr database^10,39^. Transdecoder used both datasets to predict the best ORF per starting transcript. In a few minority cases, TransDecoder preferentially chose the longer ORF over one with protein homology, we therefore wrote a custom script to select the ORF with homology in these cases. Redundancy was then removed from the dataset using cd-hit and our custom program which clusters using BLAST, in both cases the longest ORF per cluster was retained. To annotate these non-redundant sequences, we ran them through BLAST2GO which uses protein blast results to describe and assign GO-terms. This step removed a large number of sequences derived from transposases. Our final dataset consisted of 23,047 annotated cDNAs as described in the results section.

### Repeat Depletion

To repeat-deplete the Keinath *et al*. whole genome reads, each read file was run through khmer to calculate the median coverage based on kmers of 31 bp^29^. Read pairs were removed if either read had a coverage less than or equal to 1 or greater than 40. We also removed any read pairs which contained an ambiguous nucleotide (‘N’) using a python script available alongside VGW.

### Virtual Genome Walking

The Virtual Genome Walking (VGW) pipeline first divides the input genome read files into multiple sub-files, each of which will be indexed using BWA. Optionally each of these sub-files can be compressed using gzip at the expense of running speed. Once created, the index can be reused indefinitely.

On the first VGW iteration, transcript sequences are mapped to indexed reads using BWA fastmap with a minimum super maximal exact match (SMEM) of 40 bp^30^. This permits reads to map to almost all exons. The mapped reads are then extracted into separate files per starting transcript, and assembled using SOAPdenovo2 (k = 63 bp) and fermi-lite (https://github.com/lh3/fermi-lite/) with default parameters^40^. The output from both processes are assembled using CAP3 (-k 0 -p 75 -o 30 -h 80 -f 200 -g 4) with parameters that permit longer overhanging sequences. The original transcript and CAP3 output are aligned using BLASTn with a culling limit of 2 to extract assembled contigs that contain exon sequence. Finally, the extracted reads are mapped using BWA and any contigs that share sufficient paired-end read mappings with the exon-containing contigs are also retained. This ensures that any close neighbour contigs that do not contain exons are extended in the following iterations.

On all following iterations, 600 bp at the ends of each contig are mapped to the reads using a longer SMEM of 60 bp. As before, SOAPdenovo2 and fermi-lite are used to locally assemble the extracted reads. An additional elongation step is added whereby the short reads mapping to each contig are assembled using CAP3 with default parameters. These contigs are combined with the SOAPdenovo2 and fermi-lite contigs, and the final contigs from the previous iteration prior to assembly with CAP3 (overhang parameters). The exon-containing and potentially neighbouring contigs are identified as before. After each iteration, the maximum contig length and the sum lengths are compared to the previous iteration. If either of these has increased, then another iteration will be run on that transcript. The number of contigs is also compared and if it reaches a set maximum value (by default 500) or triples within a single iteration then that transcript is no longer processed. This ensures that repeat containing contigs do not disrupt the VGW process by assembling sequence from across the genome.

After the final iteration is complete, we first check that each contig appears correctly assembled. The transcript and contigs are aligned by BLASTn, identifying the contigs that contain multiple HSPs to the same exonic sequence. For each of these potentially problematic contigs, all of the previously extracted reads are mapped using BWA. At each position along the contig the coverage of paired end reads mapping with 100% identity is calculated. Any region with a coverage of 0 that is not at the ends of the contig, or has no paired end reads spanning it, is removed to divide the chimeric contig into sub contigs.

Contigs are reverse complemented according to the transcript, and each contig is compared against each other by BLASTn to search for large regions of similarity. Within each group a consensus sequence is derived by aligning the contigs using MAFFT^41^. Therefore alternative assemblies of the same locus, which may differ in the length of a repeat, do not appear as contiguous genome fragments.

The re-orientated, consensus derived contigs are ordered according to BLASTn HSPs with the original transcript. Each contig is aligned with the following contig using BLASTn to look for a potential overlap at the respective ends. If there is an overlap, then the contigs are joined using a consensus sequence of the overlap region (MAFFT). Contigs with no perceivable overlap are joined with an arbitrary string of 500 ‘N’s, generating a single genome scaffold per original transcript.

### VGW scaffold processing

Prior to analysis we parsed the VGW scaffolds according to the original transcript. We used GMAP to find exon-intron aware cDNA matches in each VGW scaffold using the starting transcript^31^. Some contigs did not contain an exon mapping to them, this appeared to be due to closely related gene copies with a shared exon sequence. VGW was walking out from two copies of near identical exons, only one of which could be successfully mapped to by GMAP. We therefore removed these non-mapping contigs, identified through the 500 ‘N’ breaks, from the scaffolds.

To identify redundant VGW scaffolds we re-mapped the transcripts using GMAP to the parsed dataset. Starting from the longest VGW scaffold, we used the GMAP GFF3 file to identify additional transcripts with overlapping exon positions. We then compared the VGW scaffolds derived from these transcripts using BLASTn, so long as all exon positions were within an HSP of at least 95% identity and at least one was positioned within an HSP of 99% identity the scaffolds were merged. We used CAP3 to assemble the contigs from all VGW scaffolds together. For any new contigs that could not be assembled this way, we first checked if they shared identity with any other contigs and if so, used MAFFT to align and generate a consensus sequence. The final collection of contigs were ordered according to how each transcript mapped, generating a single VGW scaffold per axolotl gene.

### Genome Visualization

Genome visualization was done in the R/Bioconductor package GVIZ^42^. Exon positions were identified using GMAP. Coverage was determined using all repeat-depleted reads mapped with BWA MEM and samtools depth unless otherwise stated^30,43^. To import into R, coverage and %GC means were averaged over 20 bp windows, they were then visualised in fixed 100 bp windows. For the BACs, no prior compression was required and the coverage depths were visualised in 250 bp windows. For clarity only GMAP results with more than one exon are displayed for the larger datasets of all cDNAs and the Jiang and Bryant transcripts unless otherwise stated^12,13^.

### Extending VGW contigs

To identify VGW contigs that are still extending, contigs derived from iteration 30 were aligned against contigs from iteration 31 using BLASTn. If the top hit in iteration 31 was longer, then we determined which end of the original contig had extended based on the BLASTn HSP. We examined coverage by extracting data from the samtools depth file in a 60 bp window from the end of each contig. The input cDNA transcripts were mapped against the contigs using GMAP, only the exon positions used to derive that VGW scaffold were used. Since the VGW scaffolds had been previously merged, there were some contigs with no exons mapped, these were excluded from the length analyses. These data were combined to analyse which contig ends had stopped extending, their median coverage depth and the length walked from the nearest mapped exon.

### Repeat identification and masking

The merged scaffolds from VGW, assembled chromosome 13 and 14 contigs and the BACs were processed through RepeatModeler to generate a classified consensus library of 2,103 sequences^44^. This library was used in conjunction with RepeatMasker to mask repeats in our VGW scaffolds and across the BACs^45^. The length of each repeat was determined from the GFF file, irrespective of potential overlaps.

### BAC comparison

To assess the quality of VGW assemblies we aligned the scaffolds and cDNA sequences against the 24 known BACs using BWA (Table S1)^30^. Within this BAC collection there contains an apparent recent gene duplication; EU686400 and EU686411 are 99% identical over 62 kb. Only one transcript in our collection corresponds to these two genes, and it is more similar to the EU686400 annotated transcript. We therefore excluded EU686411 from further analysis. Five of the remaining 23 BACs had no annotated transcript, although we were able to find VGW scaffolds for three of these (Table S1). Seven BACs had no corresponding transcript within our annotated CDS collection.

Percent identity values were calculated directly from the BAM files and contiguity was determined from mummer dotplots^46^.

### Human comparison and synteny

All chromosomal protein-coding transcripts from Ensembl release 89 (May 2017) were downloaded and made into a BLAST database of 80,434 cDNAs^47,48^. This database was searched against to find the human ortholog using a BLASTn e-value of 1e-10. For axolotl transcripts which identified the same human ortholog, we selected the axolotl transcript with the longest mean intron length. Information on human introns, exons and chromosomal location were extracted using the Ensembl API. Exon boundary sites were compared using a multiple alignment (muscle) of corresponding transcript sequences^49^. The same protocol was used to compare human against axolotl, as to compare human and *Xenopus tropicalis*. To ensure that we were comparing like with like, exon boundaries that were not present in the opposing species were excluded as well as genes with a mean distance greater than 50 (939 axolotl genes and 232 *Xenopus tropicalis* genes) Large-scale synteny between human and chicken was obtained directly from the Ensembl website.

### Data Availability

The VGW scaffolds, input transcripts and annotation files are available to download at figshare (https://tinyurl.com/y8gydc6n). The VGW program and accompanying python scripts are available at github (https://github.com/LooseLab/iterassemble) and include running instructions and an example dataset.

## Electronic supplementary material

Supplementary Figures and Tables
