# Publisher Correction: Virtual Genome Walking across the 32 Gb *Ambystoma mexicanum* genome; assembling gene models and intronic sequence

**DOI:** 10.1038/s41598-018-24191-8

**Published:** 2018-04-13

**Authors:** Teri Evans, Andrew D. Johnson, Matthew Loose

**Affiliations:** School of Life Sciences, University of Nottingham, Nottingham, NG7 2UH UK

Correction to: *Scientific Reports* 10.1038/s41598-017-19128-6, published online 12 January 2018

In the original version of this Article, the author Andrew D. Johnson was incorrectly indexed. This error has now been corrected.

