# Supplementary material for: Virtual Genome Walking across the 32 Gb Ambystoma mexicanum genome; assembling gene models and intronic sequence
Source: Sci Rep. 2018 Jan 12;8:618. doi: 10.1038/s41598-017-19128-6 (PMC5766544; doi:10.1038/s41598-017-19128-6)
Supplement: Supplementary file 1 — Supplementary Figures and Tables [file 41598_2017_19128_MOESM1_ESM.pdf]

Virtual Genome Walking across the 32Gb *Ambystoma mexicanum* genome; assembling gene models and intronic sequence

Teri Evans, Andrew Johnson, Matt Loose*

Teri.evans@nottingham.ac.uk
Andrew.d.johnson@nottingham.ac.uk
*Corresponding author: matt.loose@nottingham.ac.uk

Institutional address for all authors:
School of Life Sciences, University of Nottingham, Nottingham NG7 2UH

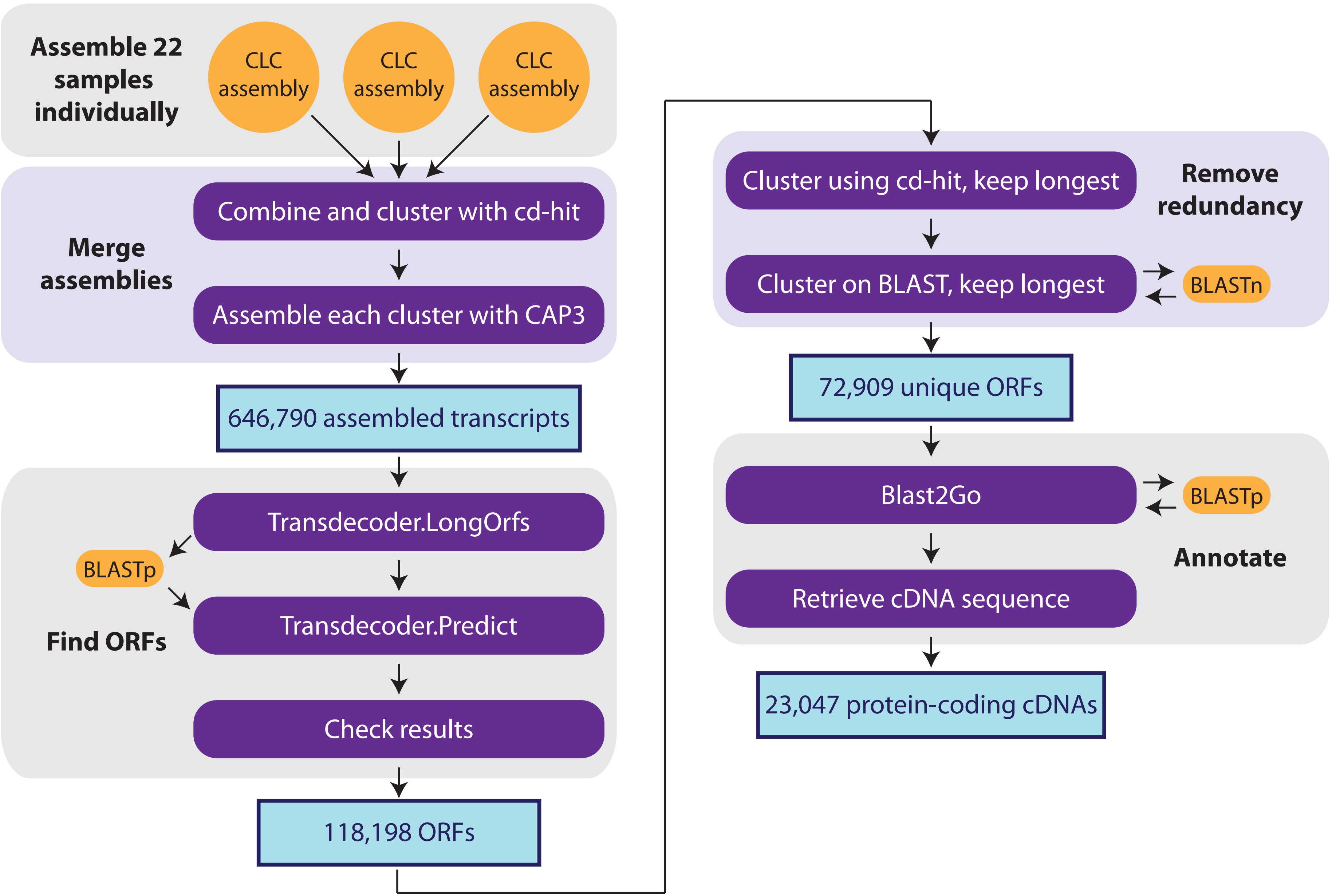

Figure S1. Transcriptome assembly pipeline. 22 RNA-seq samples of axolotl oocytes and early embryos were assembled following this protocol to form a final collection of 23,047 unique protein-coding cDNAs.

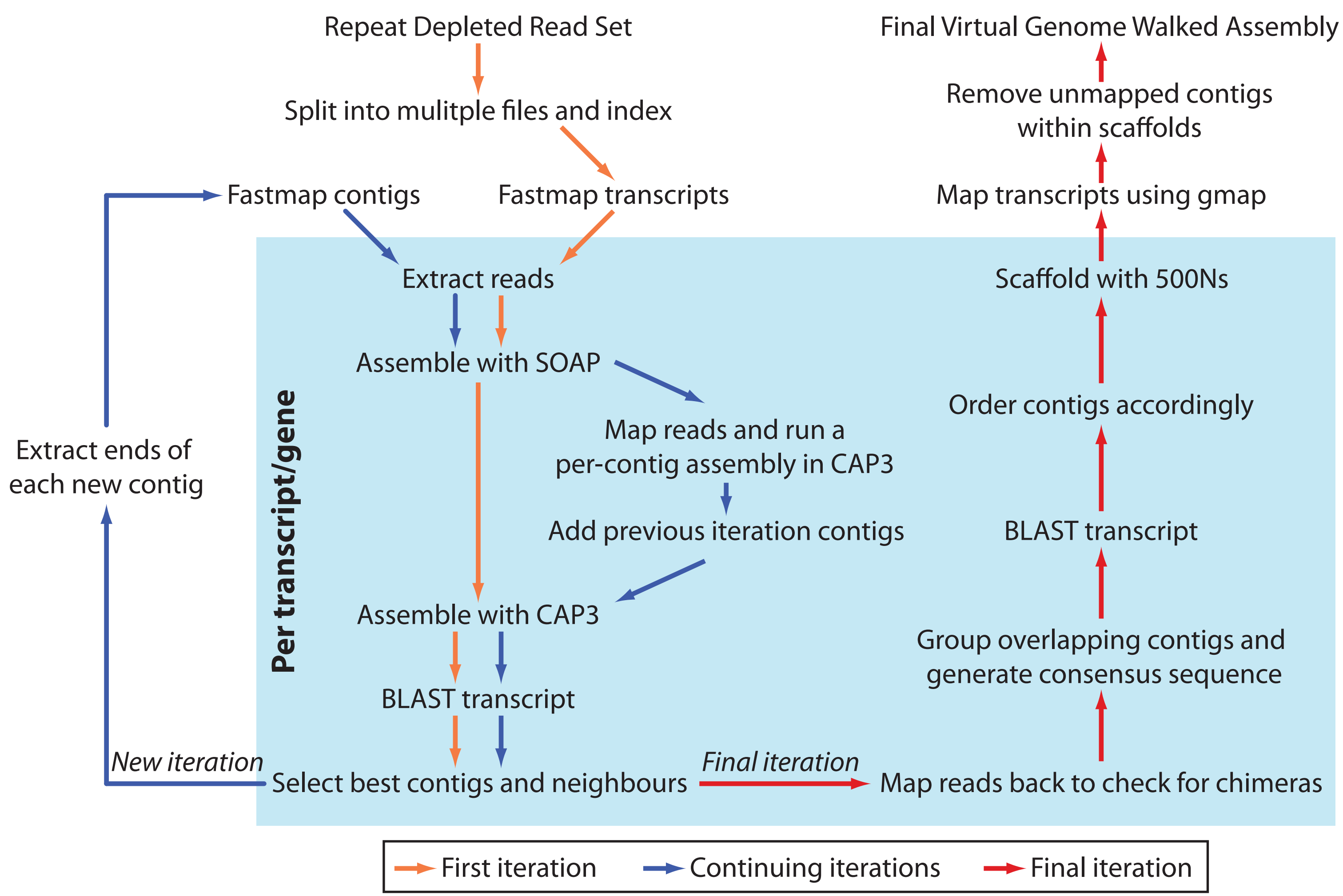

Figure S2. Detailed workflow of the Virtual Genome Walking pipeline. The process was altered depending on which iteration was ongoing (colored arrows). The steps in the blue box were run in parallel on each transcript/gene.

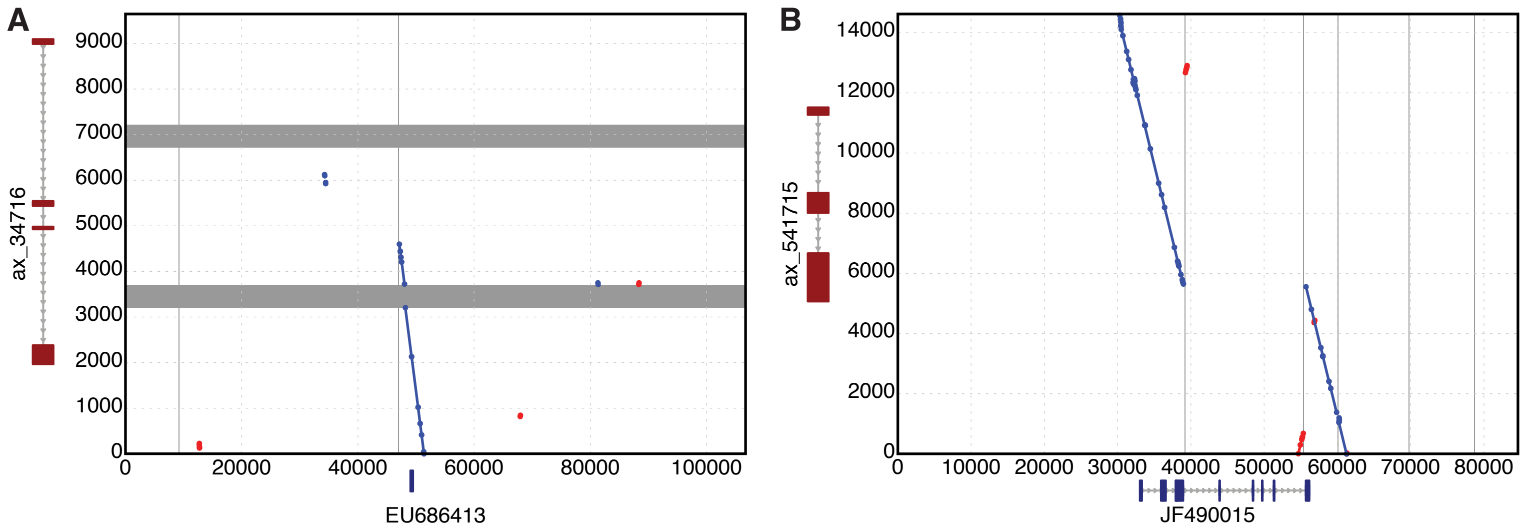

Figure S3. Dot-plots of the two VGW-BAC comparisons summarised in Figure 4C. Grey bars represent unassembled fragments and are shown to scale. The GMAP identified exons are shown alongside each input scaffold.

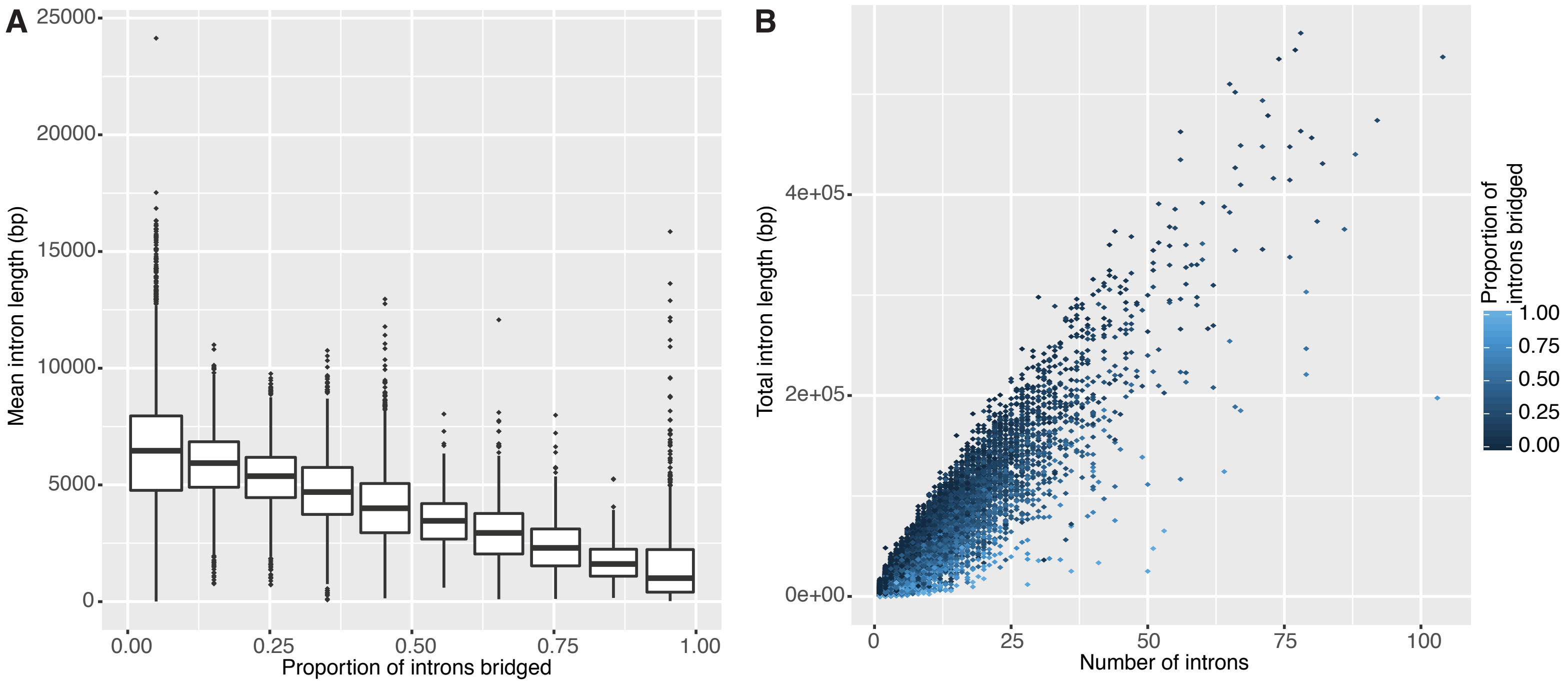

Figure S4. Axolotl intron lengths. A) Boxplots of mean intron length vs. proportion of introns able to be bridged. B) Total intron length correlates with the number of introns, the genes VGW can bridge tend to have a short total length and fewer introns.

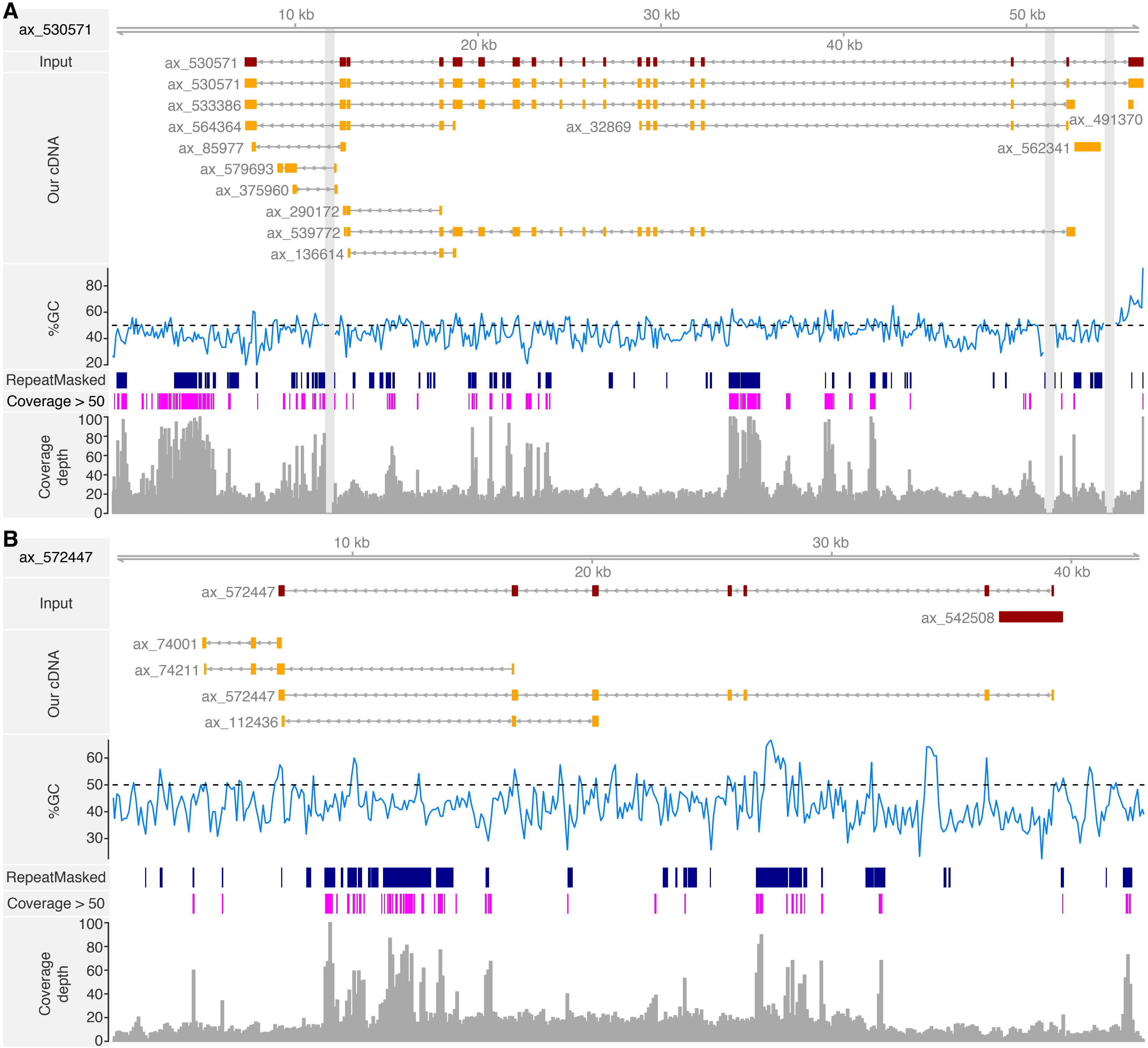

Figure S5. Examples of assembled axolotl introns. (A) The VGW output for ax_530571 with a 16Kb bridged intron and (B) ax_572447 a single contig of over 40Kb.

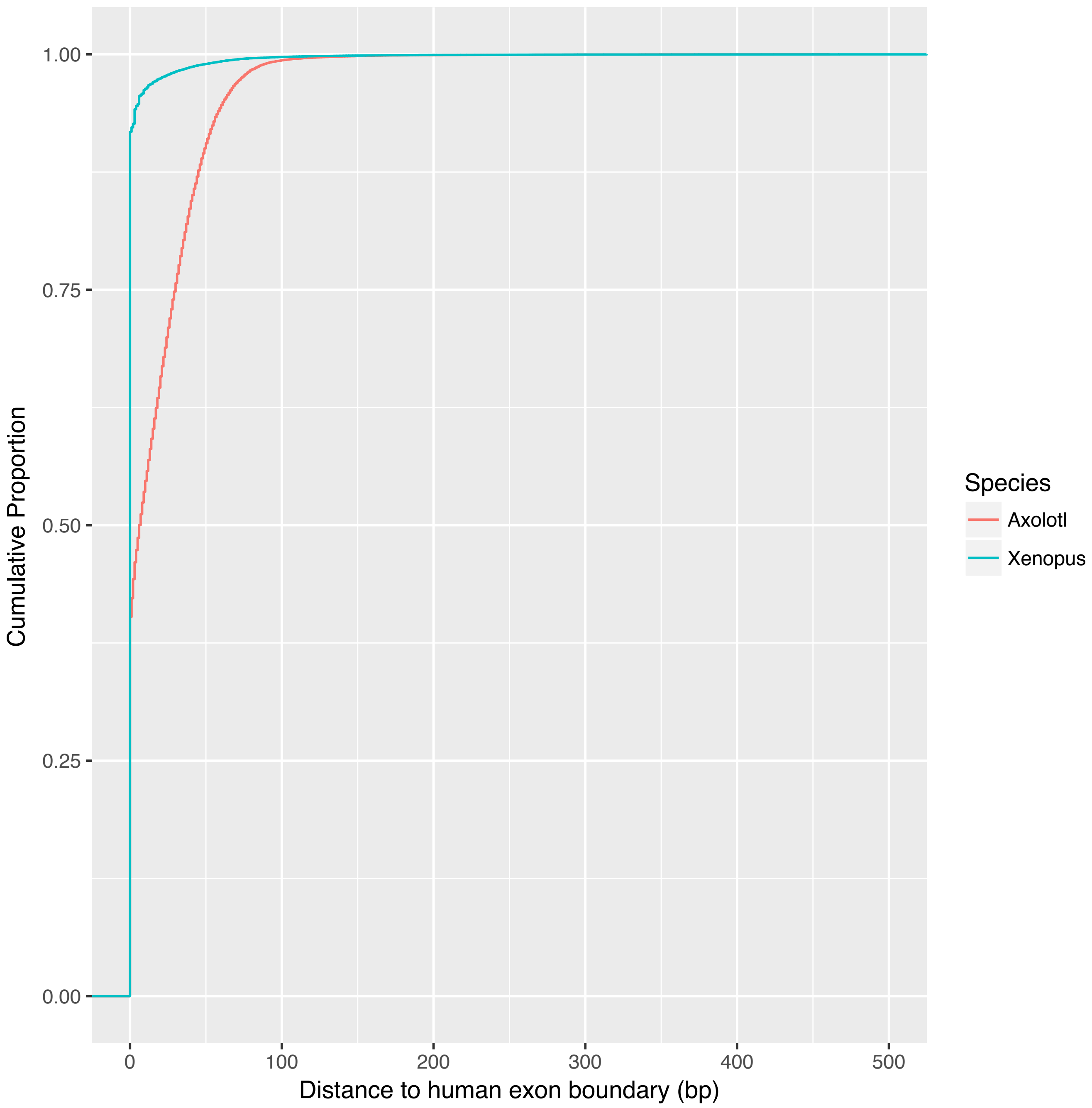

Figure S6. The exon-exon boundaries for orthologous transcripts compared to human for axolotl (red) and Xenopus (green).

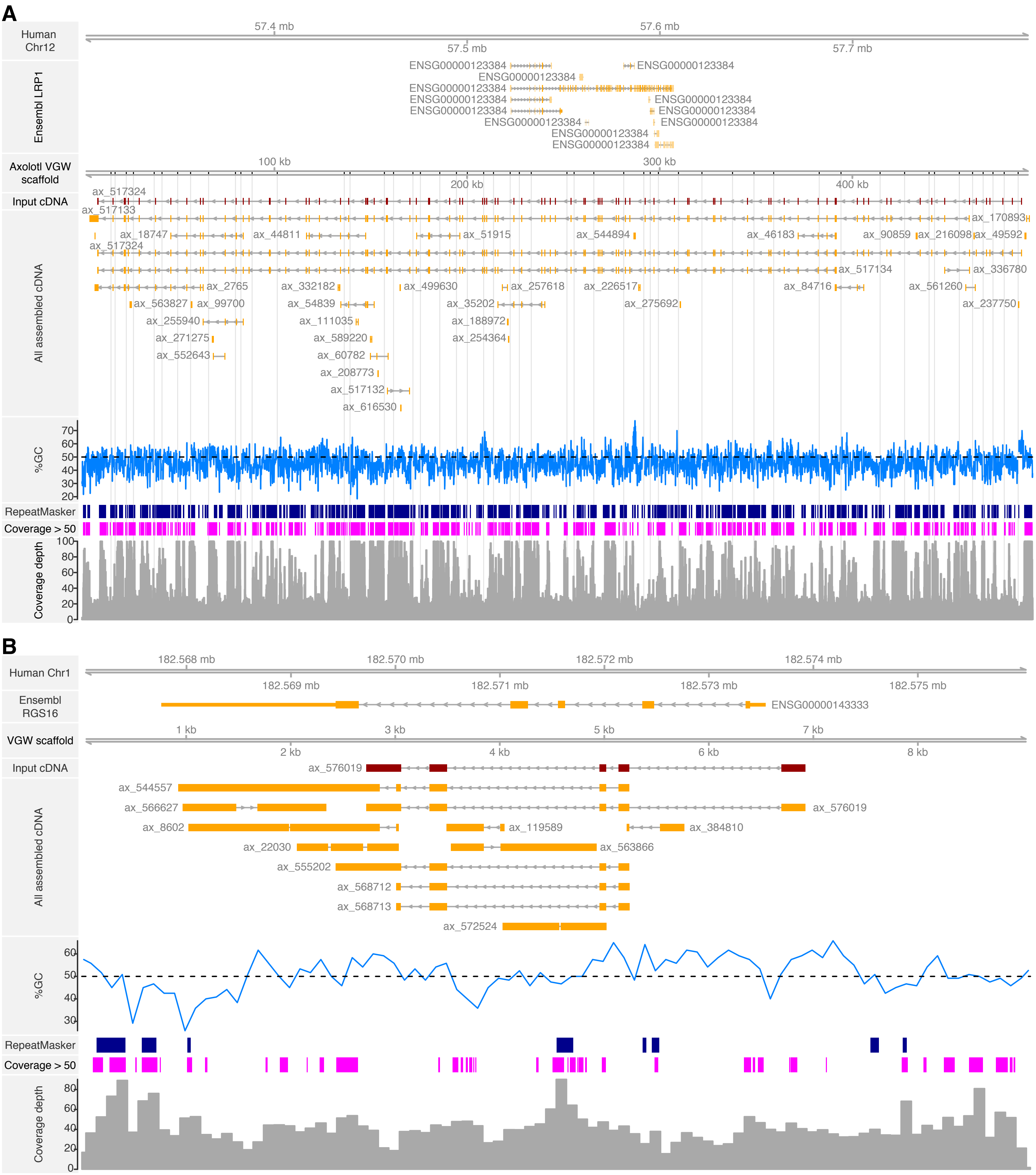

Figure S7. The VGW output for (A) ax_517324 and (B) ax_576019 compared to their human orthologs. (A) The axolotl gene is considerably larger than its human ortholog, the second example (B) shows orthologs that are similarly sized. The human and axolotl genome fragments are shown on the same scale, grey bars represent unassembled contig breaks.

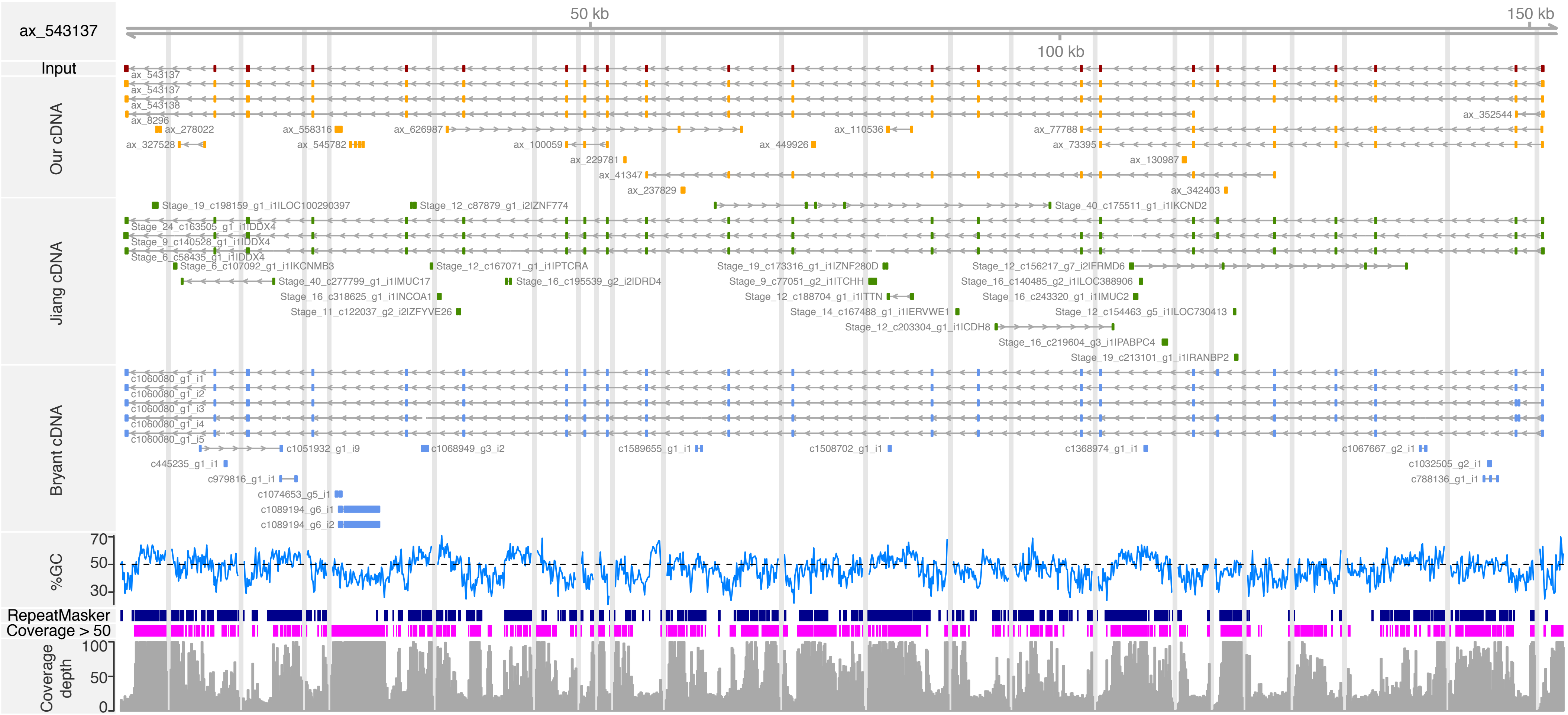

Figure S8. The VGW scaffold for DDX4 (ax_543137) is shown, vertical grey bars represent contig breaks. Transcripts assembled from our own data, alongside those from the Jiang and Bryant datasets are shown mapped to the scaffold [12,13]. This demonstrates the multiple transcript variants of DDX4 expressed in axolotl. For visual simplicity, only those transcripts with at least two exons mapped are shown.

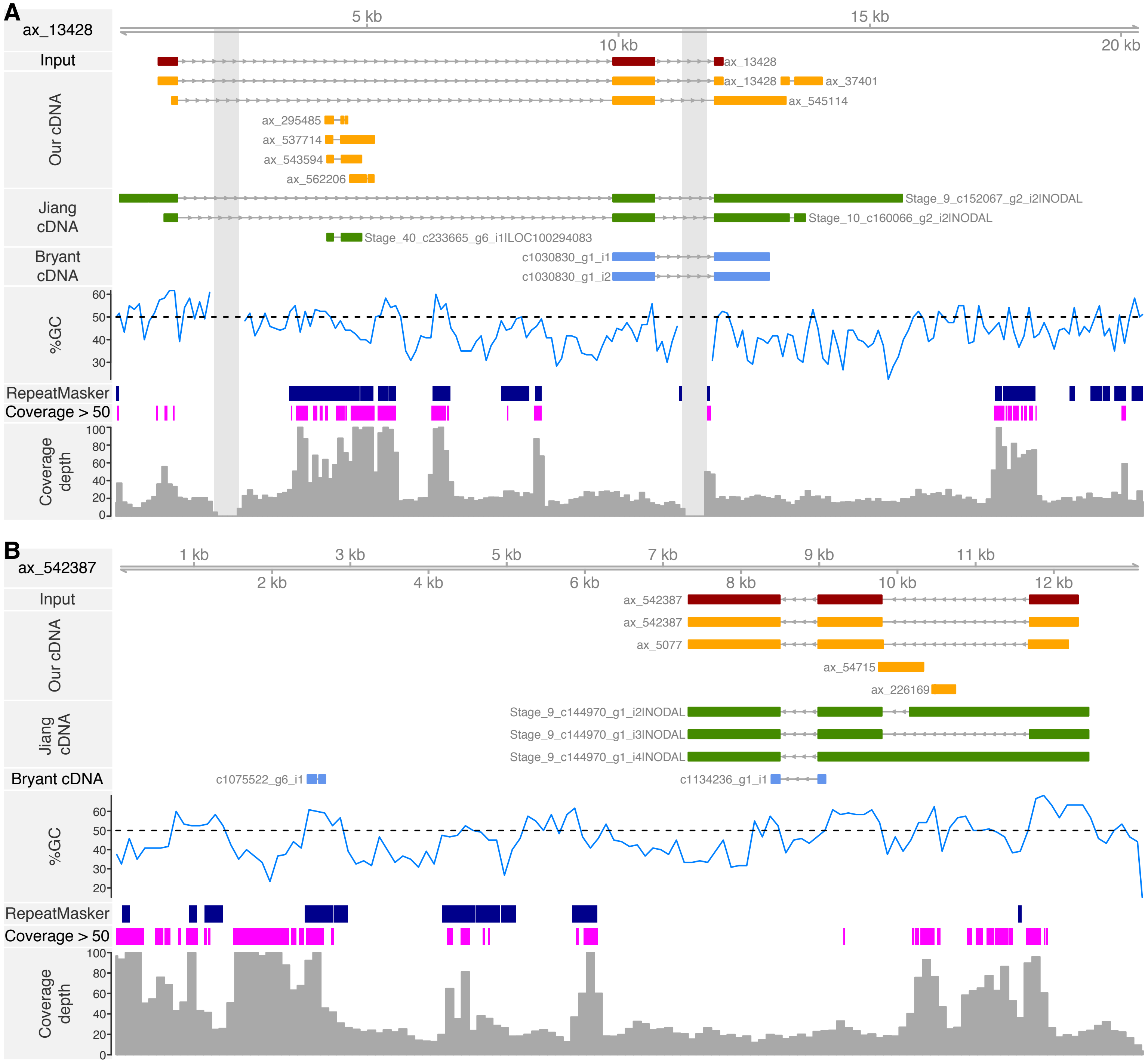

Figure S9. The VGW output for both NODAL genes. The two transcripts have assembled separate genome scaffolds and show two different gene models. Both are labeled as 'NODAL' in the Jiang dataset as they were annotated according to the single human gene [12]. Neither gene was completely assembled in the Bryant dataset [13].

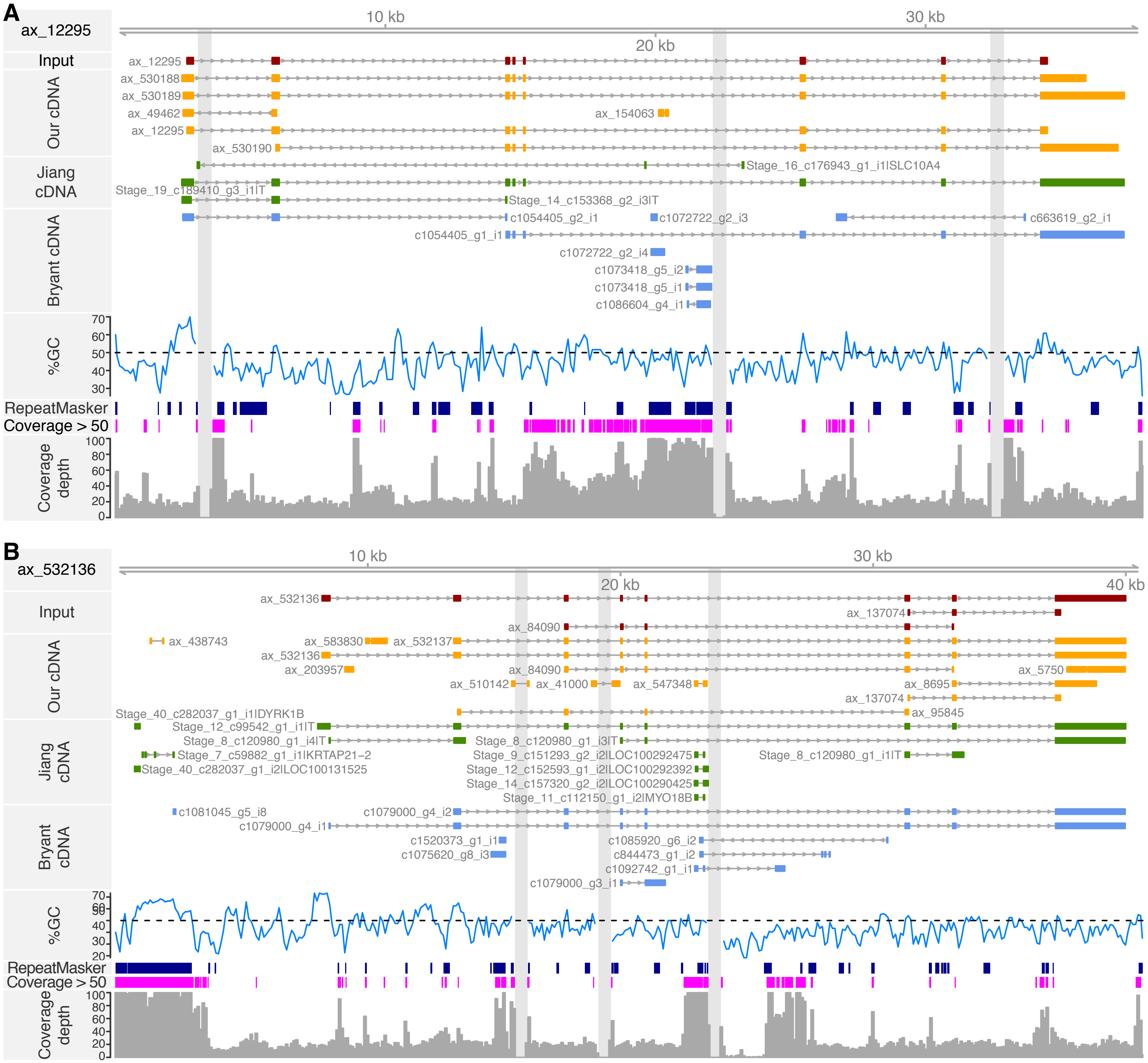

Figure S10. The VGW output for both Brachyury genes. These two transcripts have assembled different scaffolds and show two different gene models. Both are labelled as ‘T’ in the Jiang dataset as they were annotated according to the single human gene [12].

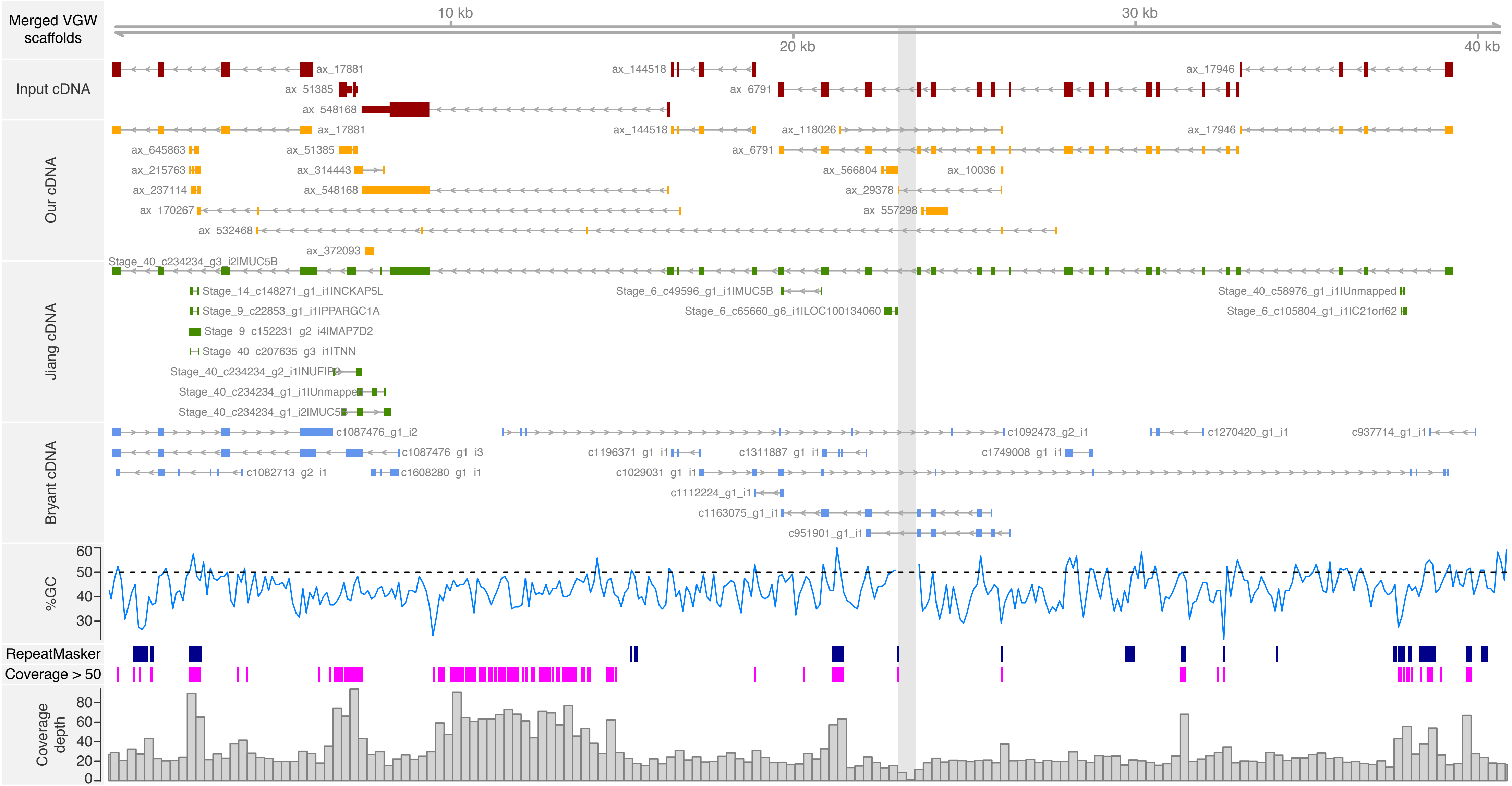

Figure S11. Three VGW scaffolds were merged, demonstrating the fragmented transcripts in our collection that are all derived from a single gene. A completely assembled transcript annotated as MUC5B was found in the Jiang dataset [12].

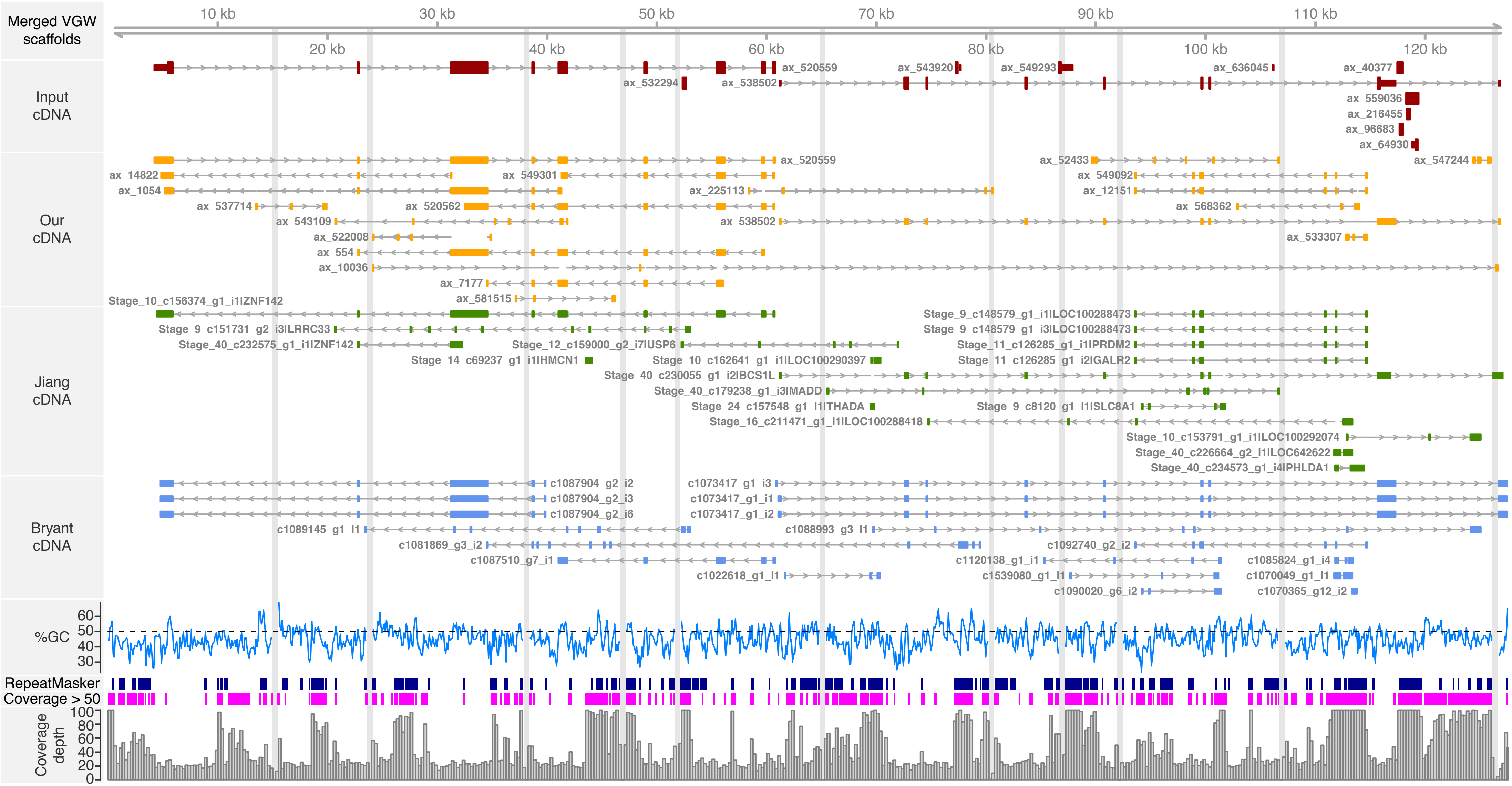

Figure S12. VGW was able to walk between these syntenic genes, ZNF142 and BCS1L. For clarity, only transcripts with at least four exons are shown in the all cDNA, Jiang and Bryant tracks [12,13].

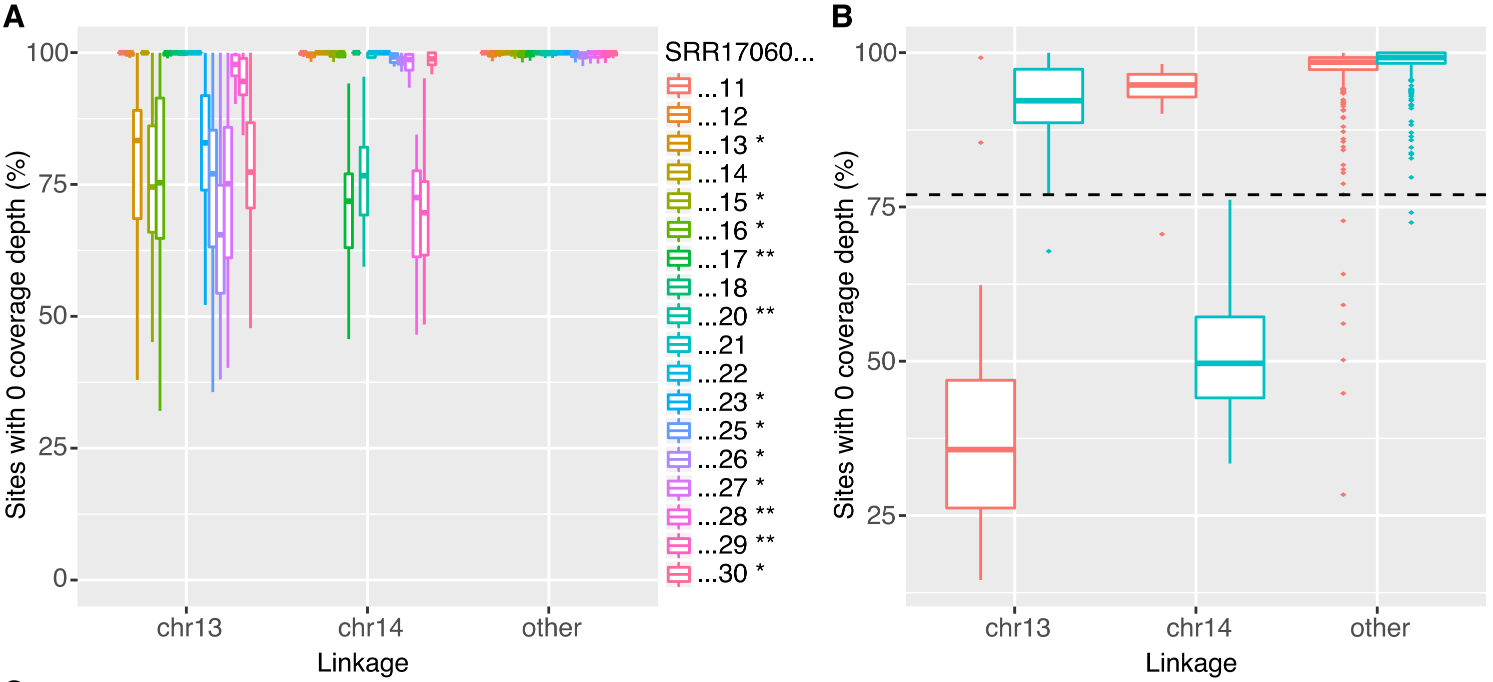

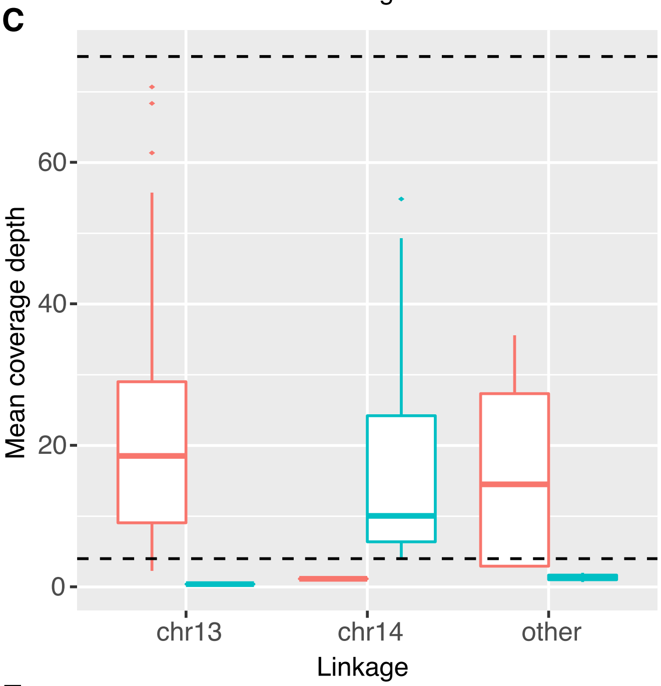

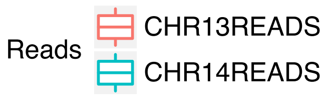

D

Linkage Group	Reads	Originally	After filtering	Remaining (%)
Chr 13	CHR13	40	32	80.0
Chr 13	CHR14	40	0	0.0
Chr 14	CHR13	19	0	0.0
Chr 14	CHR14	19	19	100.0
Other	CHR13	556	4	0.7
Other	CHR14	556	0	0.0

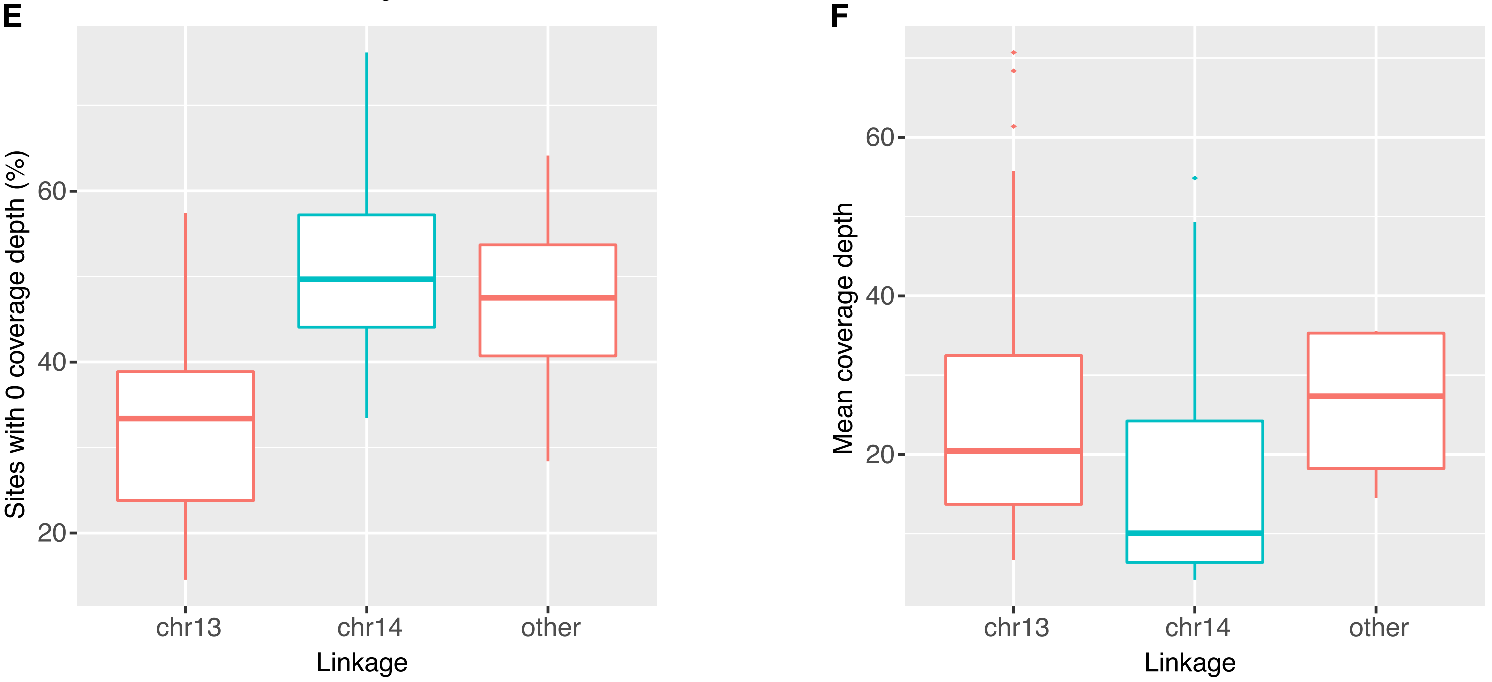

Figure S13. Filtering the Chr13 and Chr14 reads. A)The SRR libraries mapped to the linked exons, outliers are not shown for clarity. *Libraries assigned to AM13; ** libraries assigned to AM14. B) To distinguish AM13 and AM14 from other genes we removed those with more than 77% of exon sites having a coverage of 0 (dashed line). C) We also removed those with a mean depth of coverage less than 4 or greater than 75 (dashed lines). D) This removed a large proportion of genes linked to other chromosomes, and retained ~90% of the desired genes. We were unable to cleanly distinguish AM13 and AM14 from genes on other linkage groups based on sites with a coverage depth of 0 (E), or mean coverage depth (F).

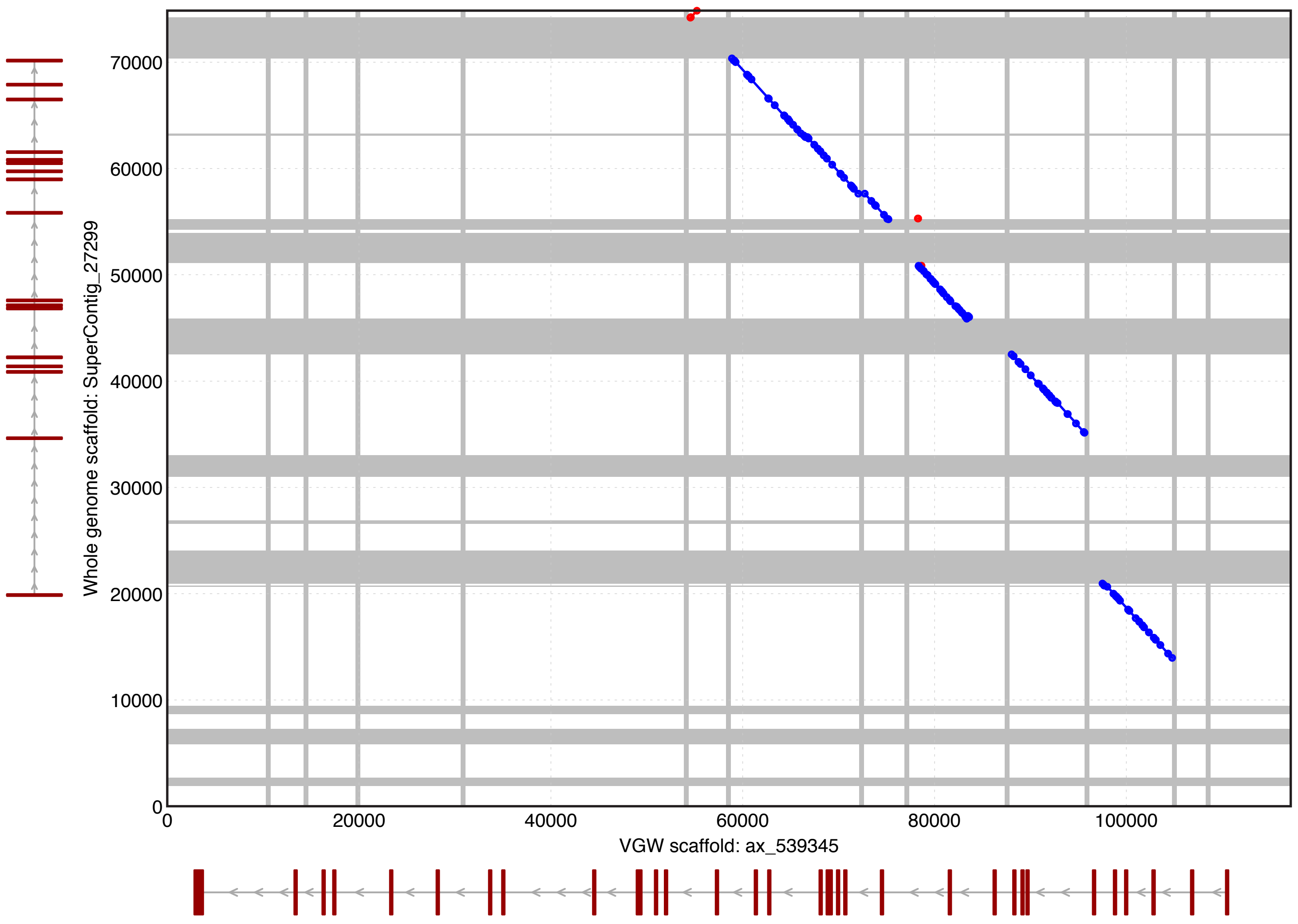

Figure S14. Dotplot comparison between one of our VGW scaffolds and one of the whole genome scaffolds. Regions with at least 100 Ns are shown in grey. The exon positions as calculated by GMAP are shown against each scaffold in red.

Table S1. The BAC results.

BAC	Gaps in BAC?	Annotated	Our cDNA	Contiguous?	Exons (on BAC)	Whole scaffold mean percent identity
JF490009	N	thrsp	-	-	-	-
JF490010	Y	nisch	ax_524095	Y	21 (1)	98.4
JF490011	Y	-	ax_528389	N**	16 (7)	97.1
JF490012	Y	arfip1	ax_540425	Y	10 (1)	98.9
JF490013	Y	adpgk	ax_1050	Y	7 (6)	97.7
JF490014	Y	col1a2	ax_526750	Y	52 (52)	99.2
JF490015	Y	c-myc	ax_541715	Y**	3 (3)	99.6
JF490016	N	nanog	ax_552852	Y	4 (4)	100.0
EU686400	N	CALR1	ax_4938	Y	9 (9)	99.7
EU686401	N	NUDT1	ax_542364	Y	7 (6)	98.2
EU686402	N	AxNovel2	-	-	-	-
EU686403	N	-	-	-	-	-
EU686404	N	HMGCR	ax_528625	Y	20 (19)	99.2
EU686405	N	AxNovel3	-	-	-	-
EU686406	N	-	ax_558334	Y	2 (2)	97.0
EU686407	N	plastin	ax_3218	Y	15 (2)	99.5
EU686408	N	P2RX3	-	-	-	-
EU686409	N	-	ax_560001	Y	1 (1)	99.3
EU686410	Y	RARRES	ax_548715	N**	6 (3)	97.9
EU686411	Y	CALR	ax_4938*	-	-	-
EU686412	Y	MIG-6	ax_565889	Y	3 (3)	99.8
EU686413	Y	Enolase 1	ax_34716	Y**	4 (1)	99.6
EU686414	Y	TIMP4L	-	-	-	-
EU686415	Y	-	-	-	-	-

*EU686411 is a recent duplication of EU686400. **The fragmented BAC is incorrectly assembled/ordered.
